# Stationary phase persister formation in *Escherichia coli* can be suppressed by piperacillin and PBP3 inhibition

**DOI:** 10.1186/s12866-019-1506-7

**Published:** 2019-06-24

**Authors:** Sandra J. Aedo, Mehmet A. Orman, Mark P. Brynildsen

**Affiliations:** 1 Department of Chemical and Biological Engineering, Princeton University, Hoyt Laboratory, 25 William Street, Princeton, NJ 08544 USA; 2 Present Address: Department of Chemical and Biomolecular Engineering, University of Houston, Houston, TX USA

**Keywords:** *E. coli*, Persisters, PBP3, Piperacillin, β-Lactam, Fluoroquinolone

## Abstract

**Background:**

Persisters are rare phenotypic variants within a bacterial population that are capable of tolerating lethal antibiotic concentrations. Passage through stationary phase is associated with the formation of persisters (type I), and a major physiological response of *Escherichia coli* during stationary phase is cell wall restructuring. Given the concurrence of these processes, we sought to assess whether perturbation to cell wall synthesis during stationary phase impacts type I persister formation.

**Results:**

We tested a panel of cell wall inhibitors and found that piperacillin, which primarily targets penicillin binding protein 3 (PBP3 encoded by *ftsI*), resulted in a significant reduction in both β-lactam (ampicillin, carbenicillin) and fluoroquinolone (ofloxacin, ciprofloxacin) persister levels. Further analyses showed that piperacillin exposure through stationary phase resulted in cells with more ATP, DNA, RNA, and protein (including PBPs) than untreated controls; and that their physiology led to more rapid resumption of DNA gyrase supercoiling activity, translation, and cell division upon introduction into fresh media. Previously, PBP3 inhibition had been linked to antibiotic efficacy through the DpiBA two component system; however, piperacillin suppressed persister formation in Δ*dpiA* to the same extent as it did in wild-type, suggesting that DpiBA is not required for the phenomenon reported here. To test the generality of PBP3 inhibition on persister formation, we expressed FtsI Ser307Ala to genetically inhibit PBP3, and suppression of persister formation was also observed, although not to the same magnitude as that seen for piperacillin treatment.

**Conclusions:**

From these data we conclude that stationary phase PBP3 activity is important to type I persister formation in *E. coli*.

**Electronic supplementary material:**

The online version of this article (10.1186/s12866-019-1506-7) contains supplementary material, which is available to authorized users.

## Background

Persisters are rare phenotypic variants within a susceptible isogenic bacterial population that have the ability to transiently tolerate lethal concentrations of antibiotics [1]. Persisters are able to resume growth upon removal of the antibiotic, giving rise to a new population with antibiotic sensitivity that is indistinguishable from that of the original population [2]. The ability of persisters to avoid eradication by antibiotics and reestablish infections has been linked to the recalcitrance of *Mycobacterium* [3], *Staphylococcus* [4], *Pseudomonas* [5], and *Candida* [6] species, as well as uropathogenic *E. coli* [7], posing significant challenges to the treatment of infections caused by these pathogens. Understanding the mechanisms that give rise to persister cell types promises to lead to more efficacious treatments for chronic, relapsing infections [8–10].

In a seminal study of persistence, Balaban and colleagues observed two fundamentally different types of persister: type I, which were generated during stationary phase, had a negligible growth-rate upon inoculation into fresh media, and whose abundance scaled with the inoculum size of stationary-phase cells; and type II that were generated continuously during growth, whose growth-rate was less than normal cells but not negligible, and whose abundance scaled with the size of the population, rather than the stationary-phase inoculum size [1]. Notably, at early times after inoculation, persisters within wild-type populations were by in large type I, whereas type II became more abundant later in growth.

A number of processes that occur during stationary phase have been linked to the formation of type I persisters [11–15], and given that one of the major physiological responses that *E. coli* mounts during stationary phase is cell wall restructuring [16–19], we sought to assess whether perturbation of cell wall biosynthesis during stationary phase impacts persister formation. We tested a panel of cell wall inhibitors on cultures undergoing the transition from exponential to stationary phase and found that piperacillin, a β-lactam that primarily targets penicillin binding protein 3 (PBP3), significantly reduced both ofloxacin and ampicillin persister levels. We investigated this phenomenon with a series of phenotypic characterizations at both the single-cell and population levels, and assessed its generality by using a genetic approach to inhibit PBP3. Overall, our data suggest that piperacillin and more generally PBP3 inhibition during stationary phase produces a phenotypic state characterized by large abundances of essential growth materials that render cells more uniformly susceptible to the bactericidal activities of β-lactams and fluoroquinolones upon introduction into fresh media.

## Results

### Treatment with some β-lactams prevents stationary-phase persister formation

To assess whether peptidoglycan physiology during stationary phase impacts persister formation, we sought conditions where treatment with cell wall inhibitors would perturb cells as they entered stationary phase but would not kill them, because cell death would have obscured any relationship between peptidoglycan restructuring and persistence. Since the transition from exponential to stationary phase begins after 4 h in our experimental conditions, we treated cultures with the inhibitors at t = 4, 5, or 6 h. Addition of 200 μg/mL of fosfomycin (MIC = 0.5 μg/mL), D-cycloserine (MIC = 32 μg/mL), or ampicillin (MIC = 4 μg/mL) resulted in cell lysis and a rapid loss of culturability, whereas treatment with mecillinam (MIC = 0.25 μg/mL) or piperacillin (MIC = 4 μg/mL) at t = 4 h or later did not lead to lysis or have an impact on culturability (Additional file 1: Figure S1). Given these data, mecillinam and piperacillin proceeded to further analyses.

**Fig. 1:**
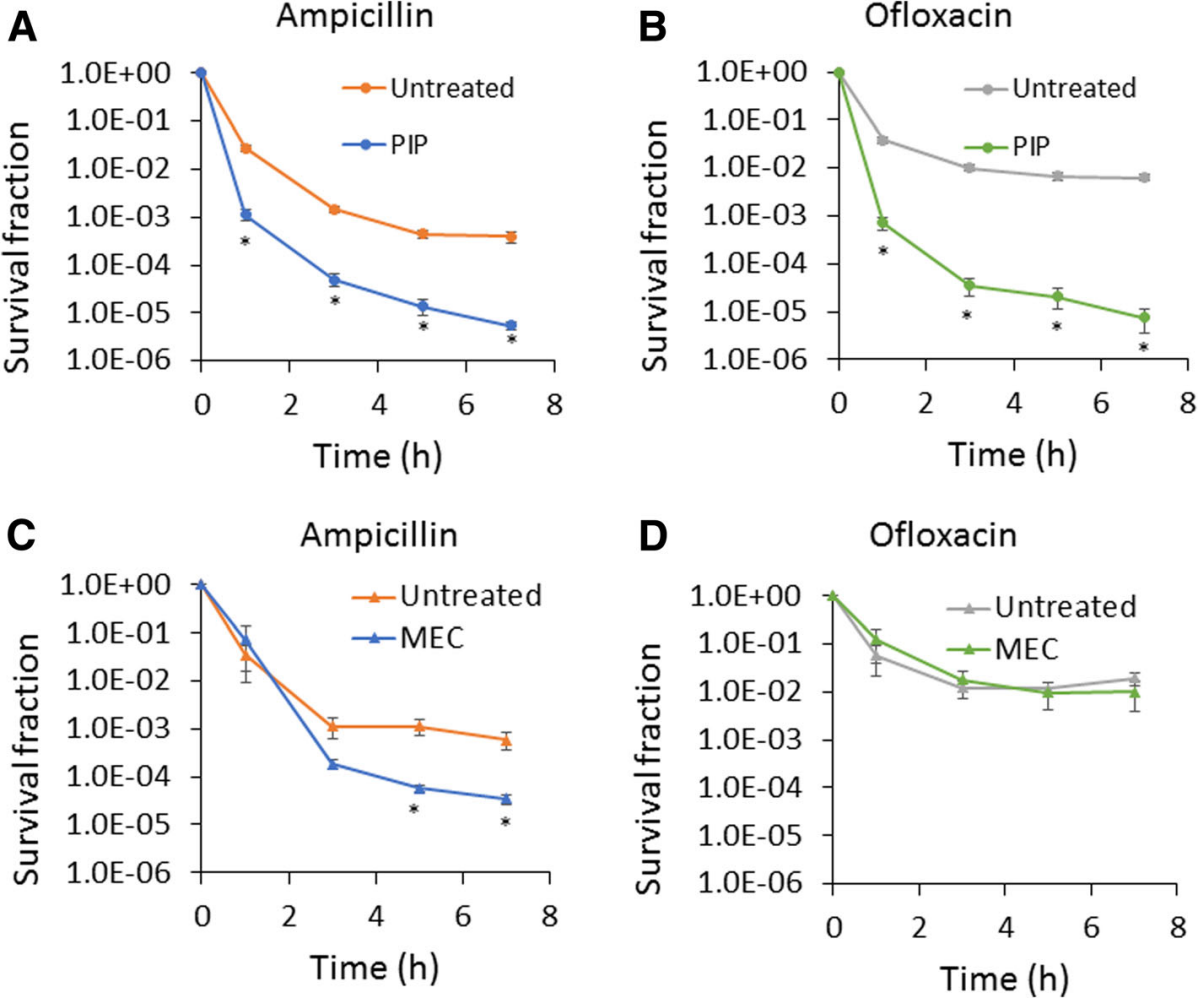
Treatment of stationary-phase cultures with specific β-lactams impairs persister formation. Cell cultures were treated with 200 μg/mL piperacillin (PIP) (**a** and **b**) or mecillinam (MEC) (**c** and **d**) at t = 4 h. Cells in control culture were treated with an equal volume of solvent, which was water (untreated). At 24 h, cultures were washed to remove chemicals and diluted in fresh LB containing 200 μg/mL ampicillin or 5 μg/mL ofloxacin. Survival fractions were monitored at the indicated time points. CFU/mL at the indicated time points are provided in Additional file 10: Figure S19. * *p* < 0.05 (t- test). Data represent three or more biological replicates. Each data point was denoted as mean ± s.e

To determine the impact of piperacillin and mecillinam on the formation of persisters in stationary phase, we treated cultures with 200 μg/mL of the inhibitors at t = 4 h, incubated them with those inhibitors until t = 24 h, and then washed off the inhibitors and conducted persistence assays in fresh media. We found that piperacillin significantly reduced both ampicillin and ofloxacin (MIC = 0.06 μg/mL) persister levels (Fig. 1a and b), whereas mecillinam significantly reduced ampicillin persister levels (Fig. 1c) but had no impact on ofloxacin persister levels (Fig. 1d). We note that persistence assays were conducted at 200 μg/mL ampicillin (50x MIC) or 5 μg/mL ofloxacin (83x MIC), which are concentrations that have been used previously to quantify persisters [2, 11, 20]. These results motivated us to further investigate the piperacillin-mediated phenotype. We performed persistence assays with another β-lactam (carbenicillin, MIC = 16 μg/mL) and fluoroquinolone (ciprofloxacin, MIC = 0.015 μg/mL), and observed similar results to those with ampicillin and ofloxacin (Additional file 2: Figure S2). This confirmed the general impact of piperacillin on the formation of persisters to these two classes of antibiotics. To assess whether these results depended on the time period that plates were incubated (16 h), we incubated plates for 48 h and found no significant difference in colonies counts (Additional file 3: Figure S3). To determine whether reduction in persister levels was associated with general growth inhibition or something specific to piperacillin, we treated cultures with chloramphenicol (MIC = 3.75 μg/mL) at 100 μg/mL at t = 4 h to inhibit protein synthesis. We found that chloramphenicol did not reduce either ampicillin or ofloxacin persister levels (Additional file 4: Figure S4).

Since inhibition of PBP3 blocks cell division and results in the formation of bacterial filaments [21, 22], we confirmed that our treatment with piperacillin during stationary phase (t = 4 h) resulted in cell filamentation (Additional file 5: Figure S5A, top panels). To further explore this phenotype, we treated cultures with piperacillin at later time points (t = 5 and 6 h) and conducted persistence assays. We found that piperacillin treatment at t = 5 h had a significant impact on ofloxacin persister levels, although it was not as pronounced as that seen for piperacillin treatment at t = 4 h (Additional file 5: Figure S5B and S5C). Further, piperacillin treatment at t = 6 h did not significantly reduce the levels of ofloxacin persisters (Additional file 5: Figure S5B and S5C). When those piperacillin cultures were observed with microscopy, we found that treatment at t = 5 h led to modest filamentation, whereas treatment at t = 6 h gave rise to stationary-phase cells with a similar morphology to that of untreated cultures (Additional file 5: Figure S5A, middle and bottom panels). Ampicillin persister levels were significantly reduced following treatment with piperacillin at t = 4, 5, and 6 h (Additional file 5: Figure S5D and S5E). These data suggest that stationary-phase formation of ampicillin and ofloxacin persisters are impacted differently by piperacillin treatment, although both are suppressed. We elected to focus on treatment with piperacillin at 4 h because it impacted the levels of persisters to different antibiotics, and thus produced physiological changes that altered the activities of two distinct antibiotic targets.

In addition to testing the impact of piperacillin at different times during transition from exponential to stationary phase, we tested the effect of different concentrations on persister levels. At 20 μg/mL piperacillin, a significant reduction in ampicillin persisters was observed, whereas at 50 μg/mL, significant reductions in persister levels were seen for both ampicillin and ofloxacin treatments (Additional file 6: Figure S6).

### Piperacillin-treated cells contain larger abundances of DNA, RNA, protein, and ATP

**Fig. 2:**
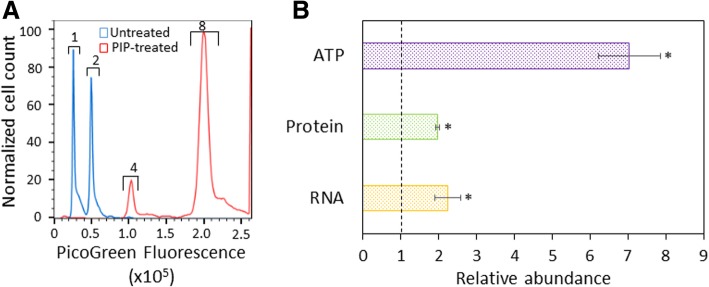
Biochemical characterization of PIP-treated stationary phase cells. Cell cultures were treated with piperacillin (PIP-treated) or water (untreated) at t = 4 h. At t = 24 h, measurements of DNA (**a**), ATP, protein, and RNA content (**b**) were carried out. **a** Cell cultures were fixed and stained with PicoGreen for DNA content and chromosome number assessment using flow cytometry. Numbers above the brackets indicate chromosome copy number. The chromosome number scale was determined with stationary-phase cell cultures of known DNA content (Additional file 7: Figure S7). **b** Cells were pelleted for RNA extraction, sonicated for protein concentration determination by the Bradford assay, or diluted to an OD_600_ of ~ 0.1 for ATP measurements using the BacTiter-Glo assay. Abundances were calculated on a per cell basis, with cell number quantified by flow cytometry, and are presented relative to untreated. * *p* < 0.05 (t-test). Data represent three or more biological replicates. Each data point was denoted as mean ± s.e

Given the larger cell size (Additional file 5: Figure S5A, top panels) observed in piperacillin-treated cultures compared to untreated controls, we sought to determine whether those cells had a larger abundance of cellular components necessary for growth. We measured DNA, RNA, protein, and ATP abundances in both piperacillin-treated and untreated cells. DNA content was measured by staining with a DNA specific dye, PicoGreen, and quantifying single-cell fluorescence using flow cytometry. Employing a reference culture with known chromosome number (Additional file 7: Figure S7), we observed that piperacillin-treated cells largely contained 4 or more chromosomes, whereas untreated cells mostly contained less than 4 chromosomes (Fig. 2a). Further, RNA, protein, and ATP levels, which are reported here as a population average on a per cell basis, were significantly higher in piperacillin-treated cells compared to untreated controls (Fig. 2b). Taken together, these data indicate that piperacillin treatment through stationary phase results in cells that are loaded with machinery necessary for growth and replication.

### Piperacillin-treated cells more rapidly and uniformly resume growth and initiate translation

**Fig. 3:**
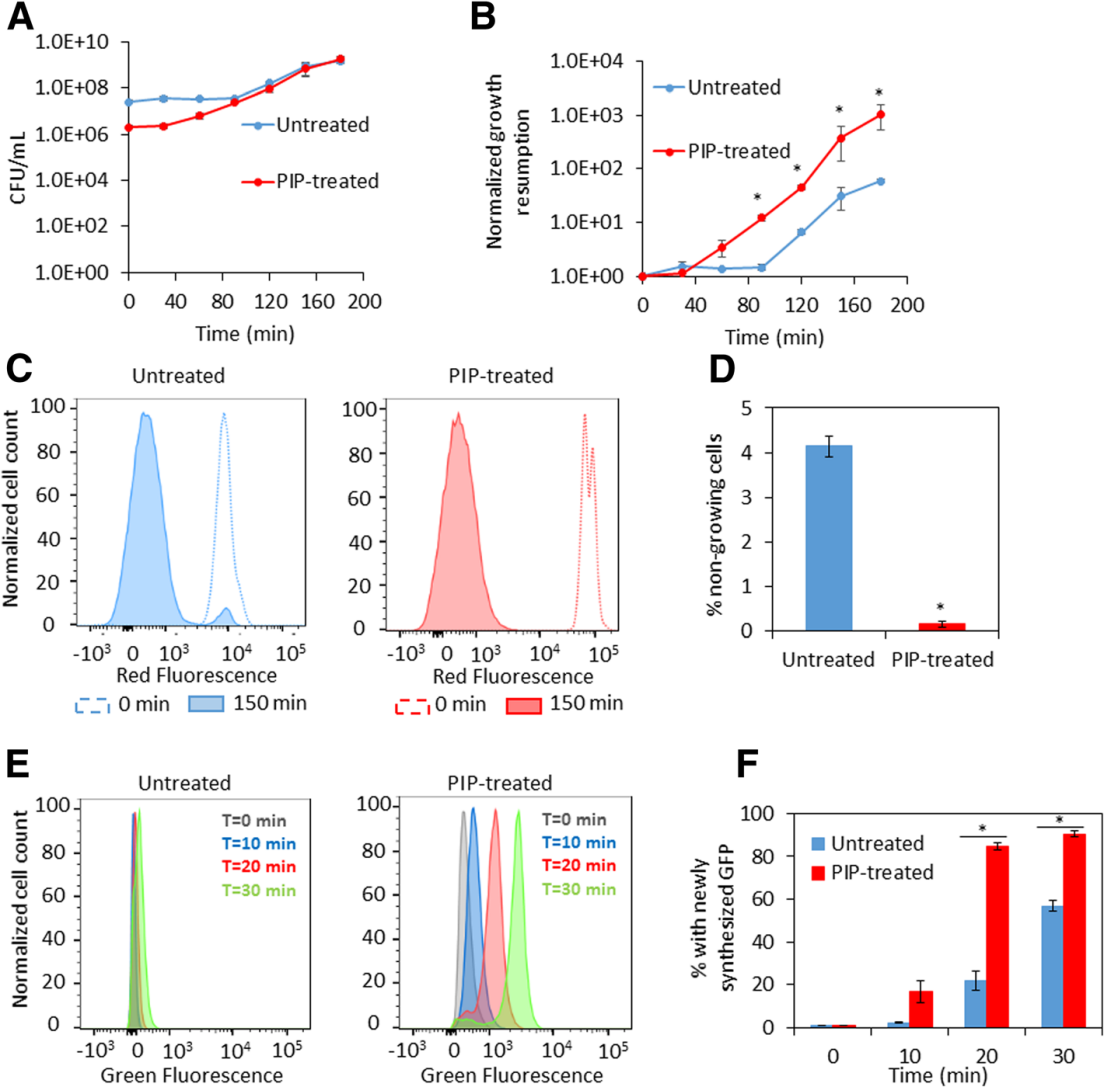
Growth resumption of PIP-treated stationary phase cells. Cell cultures were treated with piperacillin (PIP-treated) or water (untreated) at t = 4 h. At t = 24 h, measurements of growth resumption, cell division, and protein synthesis were initiated. **a** Cells were washed to remove piperacillin and diluted in fresh media. CFU/mL were monitored at the indicated time points. **b** Normalized growth resumption from A were plotted at the indicated time points. **c** Cell cultures carrying mCherry expression system were grown in LB with inducer (1 mM IPTG) until t = 24 h. At 24 h, cultures were washed and diluted in fresh LB without inducer. mCherry levels in cells at t = 0 min and t = 150 min were detected with flow cytometry (a representative replicate is shown). **d** The percentages of non-growing cells were calculated from fractions of mCherry positive cells in entire cell populations at t = 150 min. **e** Cell cultures carrying pQE-80 L-*gfp* (without IPTG) were diluted at t = 24 h in fresh LB with inducer (1 mM IPTG) for GFP expression. GFP was monitored at indicated time points with flow cytometry (a representative replicate is shown). **f** Percentage of cells with newly-synthesized GFP were plotted with respect to time. * p < 0.05 (t-test). Data represent three or more biological replicates. Each data point was denoted as mean ± s.e

Given the increased abundances of the machinery necessary for growth and replication, we assayed both piperacillin-treated and untreated cultures for growth resumption upon introduction into fresh media. We found that piperacillin-treated cultures resume growth more rapidly compared to untreated cultures (Fig. 3a and b). Significantly higher persister levels have been found in subpopulations of cells that fail to rapidly resume growth upon exposure to fresh media [1, 11, 23–26]. We hypothesized that piperacillin-treated cultures contained a lower number of cells that fail to rapidly resume growth upon inoculation into fresh media. To test this hypothesis, we measured cell division with a fluorescent reporter. In this assay, dilution of mCherry protein within cells due to cell proliferation is measured using flow cytometry. Upon removal of piperacillin (t = 0 h) and after 2.5 h of culturing in fresh media, the percentage of cells that were non-growing was significantly reduced in cultures treated with piperacillin compared to untreated cultures, which is indicative of more uniform resumption of cell division in piperacillin-treated cultures (Fig. 3c and d). To determine whether uniform growth resumption seen in piperacillin-treated cells was accompanied by a more uniform re-initiation of protein synthesis, translation of GFP was measured using flow cytometry. The results showed a significant increase in the proportion of the population that produced measurable protein in piperacillin-treated cultures when compared to untreated controls (Fig. 3e and f). These findings from single-cell assays show that stationary-phase cells from piperacillin-treated cultures resume cell division and translation more uniformly than cells from untreated cultures.

### Piperacillin-treated cells contain more PBPs and supercoil DNA more quickly

**Fig. 4:**
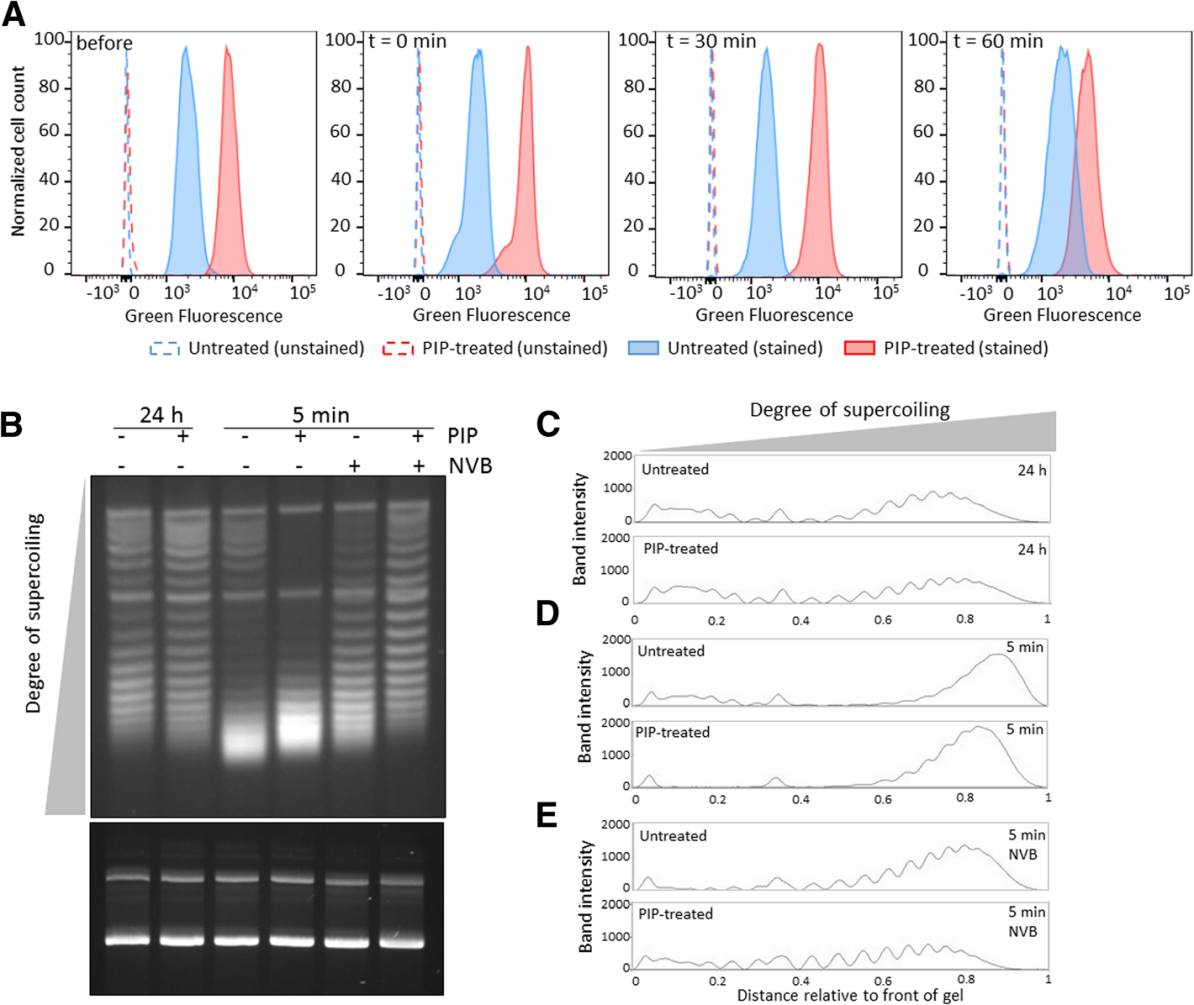
PIP-treated stationary phase cells contain more PBPs and exhibit more uniform DNA gyrase supercoiling activity upon dilution into fresh media. **a** Cell cultures were treated with piperacillin (PIP-treated) or water (untreated) at t = 4 h. At t = 24 h, an aliquot was removed for staining with Bocillin-FL (before sample). Further, at t = 24 h, cell cultures were washed to remove piperacillin and diluted in fresh LB. Aliquots were taken at the indicated time points for staining with Bocillin-FL (t = 0, 30, and 60 min). Bocillin-FL stained samples were analyzed by flow cytometry (solid lines and filled histogram) and unstained samples were used to control for autofluorescence (dashed lines and hollow histogram). Data shown correspond to one replicate of at least three biological replicates. **b** Cultures of MG1655 with pQE-80 L-kan were treated with piperacillin or water at t = 4.5 h (OD_600_ ~ 1). Piperacillin was removed at t = 24 h by washes in fresh LB, in the presence or absence of novobiocin, before dilution and incubation. Plasmid DNA was extracted at t = 24 h and after 5 min incubation in fresh media with or without novobiocin. Plasmid DNA concentration was determined and equal amounts of plasmid DNA were loaded onto an agarose gel containing chloroquine (top gel) and an agarose gel without intercalator as a loading control (bottom gel). Top and bottom gels were run for 21 h and 1 h, respectively. **c**-**e** Densitometry scans of untreated and PIP-treated samples that were either processed at t = 24 h (**c**), washed and incubated in fresh LB for 5 min (**d**), or washed and incubated for 5 min in fresh LB in the presence of novobiocin (NVB) prior to plasmid extraction (**e**). Two more replicates are presented in Additional file 8: Figure S8

To investigate the connection between piperacillin treatment in stationary phase and persistence to β-lactams and fluoroquinolones more mechanistically, we assayed for the abundance of PBPs (β-lactam primary target) and DNA gyrase activity (fluoroquinolone primary target). We hypothesized that piperacillin treatment produced cells with a greater abundance of PBPs and heightened DNA gyrase activity upon introduction into fresh media. To assess PBP abundance, we stained cultures with Bocillin-FL, which is a broad PBP-binding, fluorescent β-lactam that has been used to characterize the binding specificity of different β-lactams [27–29]. At time points, samples were fixed and stained with Bocillin-FL to provide a relative measure of PBP abundance. As depicted in Fig. 4a, piperacillin-treated cells had a higher abundance of PBPs per cell than untreated controls prior to introduction into fresh media (before), immediately upon introduction into fresh media (t = 0 min), and at later time points (t = 30 and 60 min). These data indicate that piperacillin-treated cells contain more β-lactam binding targets than untreated controls on a per cell basis (Fig. 4a). We speculate that the abundances of β-lactam binding targets in piperacillin-treated cells only begin to decrease and approach those of untreated controls by 60 min because piperacillin inhibits PBP3 irreversibly [30–33], synthesis of new PBP3 is needed for cell division [34], and division of piperacillin-treated cells does not begin until 60 min (Fig. 3a and b).

To assess DNA gyrase activity, we measured DNA supercoiling shortly after resuspension in fresh media in piperacillin-treated cultures and untreated controls. In this assay, the more highly supercoiled DNA is, the faster it will migrate in the gel [35, 36]. As shown in Fig. 4b-d and Additional file 8: Figure S8A-C and S8E-G, plasmid DNA extracted five minutes after dilution into fresh media migrates faster than plasmid DNA from 24 h stationary-phase cultures. This is the case for both untreated and piperacillin-treated cultures. However, the distribution of topoisomers from untreated and piperacillin-treated samples differs. In piperacillin-treated samples most of the slow-migrating bands (distance relative to the front of the gel of 0.0–0.3) have shifted to become fast bands, indicative of more negatively supercoiled plasmids compared to untreated controls (Fig. 4b and d and Additional file 8: Figure S8A, C, E and G). To provide evidence that the resuscitation of supercoiling is associated with DNA gyrase activity, we used novobiocin (MIC = 125 μg/mL), which inhibits the ATPase activity of GyrB, thereby preventing DNA cleavage by GyrA and in turn the ability of DNA gyrase to introduce negative supercoils [37, 38]. Our results show that incubation with novobiocin at 60 μg/mL during washes and the 5 min incubation in fresh media impairs the migration of bands toward the most supercoiled states (distance relative to the front of the gel of 0.8–1.0) (Fig. 4b and e and Additional file 8: Figure S8A, D, E and H). We note that although the impact of novobiocin was more pronounced on the piperacillin-treated cultures, plasmid supercoiling in the untreated controls was also inhibited. These data indicate that piperacillin-treated cultures exhibit heightened activity of the fluoroquinolone primary target, DNA gyrase, upon resuspension in fresh media.

### DpiA is not required for piperacillin to suppress persister formation

Previous work had established a connection between PBP3 inhibition and antibiotic sensitivity, which depended on the DpiBA two component system. Specifically, Miller and colleagues previously showed that inactivation of PBP3 induces the SOS response through the DpiBA two-component system, which leads to inhibition of cell division and aids in survival to β-lactam exposure [39]. Although the experimental conditions of Miller and colleagues varied considerably from those used here (e.g., a 25-fold lower concentration of piperacillin, temperature of 30 °C rather than the 37 °C used in this study), we sought to assess whether DpiA was involved in the ability of piperacillin to suppress stationary-phase persister formation. We note that the culturability of Δ*dpiA* populations following piperacillin treatment was significantly lower than that of comparably treated wild-type populations, whereas the culturabilities of untreated cultures of these strains were comparable (Additional file 9: Figure S9A and Additional file 10: Figure S19A). These observations suggest that DpiA is important for the survival of stationary-phase cultures treated with piperacillin, which is consistent with the findings of Miller and colleagues, though under different conditions. Because that lower culturability produced some Δ*dpiA* persistence measurements that were below our limit of detection (Additional file 9: Figure S9A and S9B), we increased the initial inoculum of Δ*dpiA* for persistence assays to match that of wild-type (see Additional file 11). For Δ*dpiA*, we observed a significant suppression in persister levels following piperacillin treatment compared to untreated controls (Additional file 9: Figure S9C and S9D) that was of similar in magnitude to that of wild-type (Fig. 1a and b). In addition, the cell sizes and morphologies of piperacillin-treated wild-type and Δ*dpiA* cultures were comparable (Additional file 5: Figure S5A and Additional file 9: Figure S9E). These data suggested that DpiA is not involved in the phenomenon under investigation here.

### Expression of an inactivated PBP3 suppresses persister levels

Relative to many other β-lactams, piperacillin targeting is highly specific for PBP3 [21, 29], however, since it can bind other PBPs, we sought to assess the generality of PBP3 inhibition on persister formation. To accomplish this, we adopted a genetic approach where a transpeptidase-negative FtsI (FtsI Ser307Ala) [40, 41] was over-expressed upon entry into stationary phase to out-compete the chromosomally-expressed native FtsI. The mutant mimics a piperacillin-impaired PBP3 [40, 41], since piperacillin inhibits PBP3 transpeptidase activity [42], and induction of the catalytically-inactive mutant (ftsI*) during stationary phase resulted in cell filamentation, which is a phenotypic outcome of PBP3 inhibition at the bacterial septum, whereas over-expression of the native PBP3 (*ftsI*) did not alter stationary-phase morphology (Additional file 12: Figure S10A). We also demonstrated that truncated mutants of FtsI and FtsI* that are devoid of their cytoplasmic and transmembrane domains [43] failed to produce filamentation (Additional file 12: Figure S10B), which suggests that the capacity of FtsI* to inhibit septation depends on its ability to localize to the membrane. We note that this truncation to FtsI has been previously shown to impair FtsI localization to the septum [43]. Confirmation of expression of FtsI_Trunc_ and FtsI*_Trunc_ from our plasmid construct was carried out by mass spectrometry (Additional file 13: Figure S11). Cultures of the wild-type strain expressing either FtsI_Trunc,_ FtsI*_Trunc_, or GFP from a low-copy number plasmid were induced overnight to ensure enough protein for mass spectrometry. Gel bands were excised between 50 and 75 kDa (expected size of truncated proteins was ~ 59 kD). The analysis yielded peptide sequences covering 29 and 57% of the full length FtsI sequence for the FtsI_Trunc_ and FtsI*_Trunc_ samples, respectively (Additional file 13: Figure S11), whereas the GFP-expressing control did not produce any peptides mapping to FtsI. In addition, the sequence corresponding to FtsI*_Trunc_ contained the expected active site mutation. Collectively, these results are consistent with FtsI* outcompeting native FtsI at the septum to inhibit PBP3 transpeptidase activity.

**Fig. 5:**
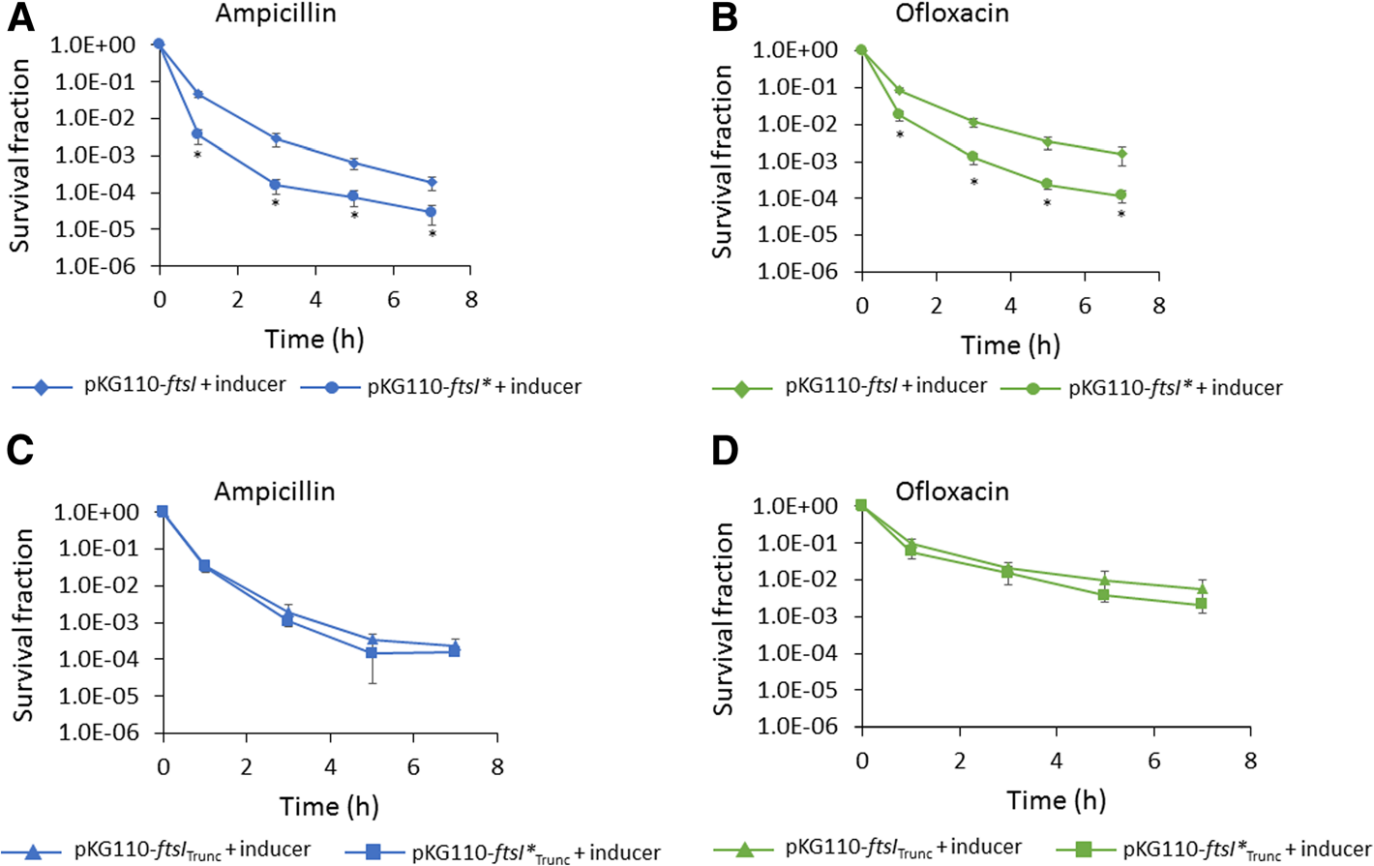
Expression of an inactivated PBP3 during stationary phase inhibits persister formation. Cultures of MG1655 carrying pKG110-*ftsI* (wild-type *ftsI*) or pKG110-ftsI* (catalytically inactive mutant) were treated with 100 μM sodium salicylate (inducer) at t = 3 h (**a** and **b**). At 24 h, cell cultures were washed to remove the inducer and diluted in fresh LB containing 200 μg/mL ampicillin (**a**) or 5 μg/mL ofloxacin (**b**). In (**c**) and (**d**) cultures of MG1655 carrying pKG110-*ftsI*_Trunc_ or pKG110-ftsI*_Trunc_ (each devoid of their transmembrane and cytoplasmic domains) were treated as in **a** and **b**, and ampicillin (**c**) and ofloxacin (**d**) persister assays were carried out. Survival fractions were monitored at the indicated time points. CFU/mL at the indicated time points are provided in Additional file 14: Figure S12E and S12H. * p < 0.05 (t-test). Data represent three or more biological replicates. Each data point was denoted as mean ± s.e

When type I persister assays were conducted, we observed significant reduction in ampicillin and ofloxacin persisters following induction of FtsI* compared to wild-type FtsI (Fig. 5a and b) and the difference was dependent on induction as demonstrated by controls without inducer (Additional file 14: Figure S12A and S12B). Further, no significant impacts on ampicillin or ofloxacin persister levels were observed following induction of FtsI*_Trunc_ compared to FtsI_Trunc_ (Fig. 5c and d)_._ These results provide evidence that septal PBP3 transpeptidase activity is important for stationary-phase persister formation. However, we note that the magnitude of reduction in persister levels was larger for piperacillin treatment compared to FtsI* expression, and that this could be associated with incomplete inhibition of PBP3 activity by FtsI* expression (native *ftsI* was still produced endogenously from the chromosome) or effects of piperacillin not associated with its primary target, such as binding to other PBPs.

## Discussion

Several processes that occur during stationary phase have been shown to impact the formation of type I persisters [11–15]. Motivated by these studies and the fact that cell wall restructuring is one of the major processes that occur during stationary phase [16–19], we investigated the impact of a series of cell wall inhibitors during stationary phase on type I persister formation. We found that piperacillin, a PBP3 inhibitor that blocks cell division and leads to cell filamentation, results in a significant decrease in both β-lactam and fluoroquinolone persister formation (Fig. 1 and Additional file 2: Figure S2). Further analyses revealed that cultures treated during stationary phase with piperacillin had more DNA, RNA, protein, and ATP than untreated controls (Fig. 2). Such traits suggest that the populations were primed for growth, and this was confirmed with single-cell assays where piperacillin-treated cultures had far fewer cells that could not rapidly resume replication and growth compared to untreated controls (Fig. 3). Looking more closely at the β-lactam and fluoroquinolone primary targets, we also demonstrated that piperacillin-treated cells had a far greater abundance of PBPs (Fig. 4a) and more rapidly reactivated DNA gyrase than untreated controls (Fig. 4b-d and Additional file 8: Figure S8A-C and S8E-G). Collectively, these data depict a phenotypic state that would be extremely vulnerable to antibiotics present in fresh media, which provides an explanation as to why such populations have so few type I persisters.

To assess the generality of PBP3 inhibition on persister formation, we used a genetic approach and over-expressed a catalytically-inactive FtsI (FtsI*) with over-expressed native FtsI to serve as a control. We observed a qualitatively similar phenomenon with this genetic system, where the inactive variant led to filamentation and lower persister levels compared to the native enzyme (Fig. 5). These data provided further evidence that PBP3 activity was important to stationary-phase persister formation in *E. coli*. In a previous study, several β-lactams and a temperature-sensitive FtsI were used to show that inhibition of PBP3 led to induction of the SOS response through increased expression of DpiA [39]. Miller and colleagues described this as a protective effect that explained why wild-type could survive four times as long as Δ*recA*, Δ*sulA*, and Δ*dpiA* in the presence of 3 μg/mL ampicillin. Further, they mentioned that overnight exposure to 2 μg/mL piperacillin reduced the culturability of Δ*dpiA* 10-fold more than it did for wild-type. We also found a reduction in the culturability of Δ*dpiA* compared to wild-type when both were incubated with piperacillin overnight; although the magnitude we observed was closer to 100-fold and likely the result of the higher concentration we used (200 μg/mL piperacillin). Interestingly, we found that stationary-phase inhibition of PBP3 increased the susceptibility of cultures to ampicillin, as well as other antibiotics, when they were inoculated into fresh media (Figure 1AB, Fig. 5, and Additional file 2: Figure S2). Further, we found that DpiA was not involved in that effect with the use of Δ*dpiA* (Additional file 9: Figure S9). These differing impacts of PBP3 inhibition on antibiotic susceptibility likely originate from the different β-lactam concentrations used (2–3 μg/mL compared to 200 μg/mL) or variations in the culturing and treatment conditions (e.g., temperature, growth phase). However, a unifying theme of these works is that modulation of PBP3 activity will influence the effectiveness of antibiotics on *E. coli* populations.

Previously, our group had shown that inhibition of stationary-phase respiration by potassium cyanide (KCN) or transfer to an anaerobic environment, suppressed type I persister levels [11]. Similar to the piperacillin-treated cultures here, cells from respiratory-inhibited populations more uniformly resumed growth and translation than untreated controls. In addition, respiratory-inhibited cultures were larger, had more protein per cell, and more intact RNA than untreated controls. Additional experimental evidence pointed toward a reduction in self-digestion during stationary phase, which rendered cells readily capable of resuming growth. Given the phenotypic parallels between non-respiring, stationary-phase populations and the phenomenon described here, we measured respiration in piperacillin-treated, stationary-phase populations and found them to be comparable to untreated controls (Additional file 15: Figure S13A). We also measured RNA integrity and protein degradation and found them to be comparable between piperacillin-treated and untreated cultures (Additional file 15: Figure S13B-D). Thus, the higher abundances of RNA and protein in piperacillin-treated populations does not correspond to a reduction in digestion of those cellular components, but rather, is likely to be associated with their much larger cell size (Additional file 5: Figure S5A, top panels). Collectively, these data concur with our previous study where respiration was found to be necessary for the majority of type I persister formation but not sufficient, since a number of mutants (e.g., Δ*dksA*, Δ*relA*Δ*spoT*) had reduced persister levels, yet respired normally [11].

Given the data presented here and that from previous studies [9, 11, 14, 24, 26, 44–46], we hypothesize that reductions in type I persister levels will occur for any perturbation that places stationary-phase populations in a phenotypic state where more cells have sufficient abundances of cellular components required for growth resumption. In the study presented here, piperacillin-treated cells were larger, with a corresponding increase in a number of important cellular quantities (Fig. 2). With their larger size, we speculate that piperacillin-treated populations had fewer cells with low levels of components that are needed for growth resumption than untreated cultures. As mentioned above, respiratory-inhibited populations also produced larger cells, and those cells displayed reduced hallmarks of self-digestion, which would also serve to maintain cellular components at levels needed for growth resumption [11]. However, we note that some means of changing cell size, such as DNA damage [47, 48], are unlikely to produce physiologies that can readily resume growth. Mordukhova and Pan observed that growth to stationary phase at 42 °C increased type I persister levels that were measured in minimal media with ampicillin and ofloxacin at 37 °C [14]. MetA, which is a methionine biosynthesis enzyme, is essential for growth in minimal media and it is prone to aggregation at elevated temperatures. Mordukhova and Pan found that expression of a heat-stabilized variant of MetA led to lower persister levels than a strain expressing the native MetA when cultures were grown to stationary phase at 42 °C. That data suggests that for populations cultured at 42 °C, substitution of MetA with a heat-stabilized variant produces a population with more cells with sufficient quantities of functional MetA for growth resumption, and thus lower persister levels. Peters and coworkers observed in *Bacillus subtilis* that modest knockdowns of essential genes using CRISPRi led to populations with significantly delayed growth lags when introduced to fresh media [49]. The authors showed that the growth lags were not associated with maximal growth-rates, but rather a higher proportion of non-growing cells [49]. Although Peters and colleagues did not measure persistence, their study suggests that reducing the expression of an essential gene in stationary phase can produce larger subpopulations of non-growing cells, and it has been shown by others that non-growing subpopulations house the majority of persisters in growing cultures [1, 25].

## Conclusions

Type I persisters are largely generated in stationary phase [1], which suggests that the processes bacteria execute while sensing and responding to nutrient depletion play important roles in persister formation. This postulate is supported by the work presented here, where interfering with peptidoglycan biosynthesis during stationary phase resulted in large reductions to type I persister levels, and a previous study that demonstrated that inhibition of stationary-phase respiration largely reduced type I persister formation [11]. We hypothesize that additional stationary-phase processes are required for type I persister formation, and that they might center on rendering bacteria with insufficient levels of one or more cellular components required for rapid growth resumption upon the introduction of fresh nutrients. Knowledge of those processes and components will facilitate greater understanding of persistence and growth resuscitation in bacteria, as well as provide additional targets for the development of anti-persister therapies.

## Methods

For a detailed description of the materials and methods used please see Additional file 11: Supplemental Methods. Bacterial strains used in this study were derived from *E. coli* MG1655 (ATCC 700926) [50] and are listed in Additional file 16: Table S1. Plasmids and DNA oligonucleotides are listed in Additional file 16: Table S1 and Additional file 17: Table S2 respectively. Additional file 18: Figure S14 summarizes the workflow of the experiments performed. Antibiotic minimum inhibitory concentrations (MIC) for MG1655 were determined by the 2-fold serial microdilution method as described elsewhere [51] and are provided in Additional file 19: Figure S15.

Chromosome staining was carried out using PicoGreen reagent. ATP content was measured using BacTiter-Glo Microbial Cell Viability Assay (Promega). Protein content was determined by the Bradford method. Total RNA was purified with RNeasy extraction kit (Qiagen) and, when indicated, analyzed with a bioanalyzer using an RNA 6000 Nano kit (Agilent Technologies, Inc., Santa Clara, CA) as described previously [11]. Enumeration of cell counts was carried out by flow cytometry using SPHERO AccuCount Fluorescent Particles at a concentration of ~ 1 X 10^6^ particles/mL. Cell division assays were carried out by measuring dilution of a fluorescent protein using flow cytometry. Protein synthesis was determined by measuring expression of green fluorescent protein using flow cytometry. PBPs were labeled with Bocillin-FL and their abundance analyzed by flow cytometry. DNA gyrase supercoiling activity was measured by a plasmid DNA supercoiling assay [36]. Protein degradation was assayed using fluorescent proteins and flow cytometry. Cell respiration was determined measuring dissolved oxygen in cell cultures. Expression of FtsI_Trunc_ and FtsI*_Trunc_ proteins was confirmed by mass spectrometry (Additional 13: Figure S11).

## Additional files

Additional file 1: **Figure S1.** Cell wall integrity and culturability following treatment with cell wall inhibitors. Cell cultures were treated with 200 µg/mL of piperacillin (PIP) , mecillinam (MEC), fosfomycin (FOS), D-cycloserine (CYS) or ampicillin (AMP) at t= 4 h (A and B), 5 h (C and D) or 6 h (E and F), as indicated by the arrows in each plot. Cells in control culture were treated with an equal volume of solvent, which with these antibiotics was water (untreated). OD_600_ (A, C and E) and CFU per mL (B, D and F) were monitored at the indicated time points. LOD: Limit of detection of the assay. Data represent at least three biological replicates. Each data point was denoted as mean ± s.e. (TIF 216 kb)

Additional file 2: **Figure S2.** Carbenicillin and ciprofloxacin persister assays. Cell cultures were treated with 200 µg/mL piperacillin (PIP) at t= 4 h. Cells in control culture were treated with an equal volume of water (-). At 24 h, cultures were washed to remove chemicals and diluted in fresh LB containing 200 µg/mL carbenicillin (CAR) or 1 µg/mL ciprofloxacin (CIP). Survival fractions (A and C) were monitored at the indicated time points. CFU/mL are provided (B and D). * *p*< 0.05 (t- test). Data represent at least three biological replicates. Each data point was denoted as mean ± s.e. (TIF 165 kb)

Additional file 3: **Figure S3.** Longer incubation of agar plates following persister assays. Persister assays were carried out as described in Figure 1. Colonies were counted after 16 and 48 h of incubation of agar plates at 37 °C. Survival fractions (A and C) and CFU/mL (B and D) are provided. LOD: Limit of detection of the assay. * *p*<0.05 (t-test). Data represent at least three biological replicates. Each data point was denoted as mean ± s.e. (TIF 165 kb)

Additional file 4: **Figure S4.** Treatment of early stationary-phase cultures with chloramphenicol. Cultures were treated with chloramphenicol at t = 4 h. After 20 h of incubation (t = 24 h), cells were washed to remove chloramphenicol and persister assays were carried out in fresh media. Survival fractions during 7 hours of ampicillin (A) or (B) ofloxacin challenge are provided. Data represent at least three biological replicates. Each data point was denoted as mean ± s.e (TIF 104 kb)

Additional file 5: **Figure S5.** . Piperacillin treatment during transition to stationary phase and its impact on persister formation. Cell cultures were treated with 200 µg/mL piperacillin (PIP) at t= 4, 5 or 6 h. Cells in control culture were treated with an equal volume of water (untreated). Cells were fixed at t = 24 h before washes, and imaged using a brightfield microscope (A). Cell cultures were washed to remove chemicals and diluted in fresh LB containing 200 µg/mL ampicillin or 5 µg/mL ofloxacin. CFU levels were monitored at the indicated time points. Data denoted as survival fraction (B and D) and CFU/mL (C and E). * *p*<0.05 (t-test). Data represent at least three biological replicates. Each data point was denoted as mean ± s.e. (TIF 342 kb)

Additional file 6: **Figure S6.** Impact of different concentrations of piperacillin on persister levels. Cultures at early stationary phase (t = 4 h) were treated with either 20 µg/mL piperacillin (A and B) or 50 µg/mL piperacillin (C and D). Control (untreated) was treated with equal volume of water. At t = 24 h, cells were washed, diluted into fresh media and treated with either 200 µg/mL ampicillin or 5 µg/mL ofloxacin. Survival fractions were monitored for 7 h. * p<0.05 (t-test). Data represent at least three biological replicates. Each data point was denoted as mean ± s.e. (TIF 151 kb)

Additional file 7: **Figure S7.** Cultures to identify chromosome number. An overnight culture of MG1655 was diluted 10^7^-fold into LB containing 0.2% glucose (Glc) and incubated for 144 h. One mL of sample was taken, fixed, and stained with PicoGreen at 24, 48, 72 and 144 h. Representative experiment of three biological replicates. (TIF 144 kb)

Additional file 8: **Figure S8.** Additional replicates of the DNA gyrase supercoiling assay during growth resumption. Cultures of MG1655-pQE-80L-kan were treated with piperacillin (PIP) or water (untreated) at t = 4.5 h (OD_600_ ~ 1). At t = 24 h, piperacillin (PIP) was removed by washes in fresh LB before dilution and incubation for 5 min. Where indicated novobiocin (NVB) was added to the washes and in fresh dilution media for incubation for 5 min. Plasmid DNA was extracted at t = 24 h and after 5 min incubation in fresh media. Equal amounts of plasmid DNA were loaded onto an agarose gel containing chloroquine (Top gel) (A and E) and an agarose gel without intercalator (loading control) (Bottom gel) (A and E). Chloroquine-containing gel and gel without intercalator were run for 23 h and 1 h, respectively. Densitometry scans of untreated and PIP-treated samples that were either processed at t = 24 h (B and F), washed and incubated in fresh LB for 5 min (C and G), or washed and incubated for 5 min in fresh LB in the presence of novobiocin (NVB) (D and H) prior to plasmid extraction. Gel images correspond to 2 biological repeats. (TIF 426 kb)

Additional file 9: **Figure S9.** DpiA deletion does not alter the impact of piperacillin on persister levels. MG1655Δ*dpiA*::kan cultures were treated with 200 µg/mL of piperacillin at t = 4 h. Control cultures were treated with an equal volume of solvent (water). At t = 24 h, cell cultures were washed to remove chemicals and diluted 100-fold in fresh LB containing 200 µg/mL ampicillin (AMP) or 5 µg/mL ofloxacin (OFL). (A) Survival fractions are shown at the indicated time points. (B) CFU levels are provided. At t = 24 h, cell cultures were washed to remove chemicals and diluted 10-fold in fresh LB containing 200 µg/mL ampicillin (AMP) or 5 µg/mL ofloxacin (OFL). (C) Survival fractions are shown at the indicated time points. (D) CFU levels are provided. (E) MG1655Δ*dpiA*::kan cultures were treated with piperacillin at t = 4 h. At t = 24 h, cells were fixed for microscopy analysis. * *p*<0.05 (t-test). Data represent three or more biological replicates. Each data point was denoted as mean ± s.e. (TIF 286 kb)

Additional file 10: **Figure S19.** Culturability data from stationary-phase treatment with PIP and MEC. Cell cultures were treated with 200 µg/mL piperacillin (PIP) (A) or mecillinam (MEC) (B) at t= 4 h. Cells in control culture were treated with an equal volume of solvent (water). At 24 h, cell cultures were washed to remove chemicals and diluted in fresh LB containing 200 µg/mL ampicillin (AMP), 5 µg/mL ofloxacin (OFL), or water (-). CFU levels were monitored at the indicated time points. Data represent three or more biological replicates. Each data point was denoted as mean ± s.e. (TIF 136 kb)

Additional file 11: Supplemental Methods. (ZIP 423 kb)

Additional file 12: **Figure S10.** . Expression of FtsI* resulted in filamentation whereas expression of FtsI, FtsI_Trunc_, and FtsI*_Trunc_ did not. Cultures of MG1655 carrying pKG110-*ftsI* (A, left), pKG110-ftsI* (A, right), pKG110-*ftsI*_Trunc_ (B, left), or pKG110-ftsI*_Trunc_ (B, right) were grown for 24 h. At t = 3 h, sodium salicylate (100 µM) was added to induce expression of ftsI, ftsI*, *ftsI*_Trunc_ or ftsI*_Trunc_ from plasmid. At 24 h incubation, cells were fixed for microscopy analysis. . (TIF 428 kb)

Additional file 13: **Figure S11.** Confirmation of FtsI_Trunc_ and FtsI*_Trunc_ expression by mass spectrometry. Cultures of MG1655 carrying pKG110-*ftsI*_Trunc_, pKG110-ftsI*_Trunc_, or pKG110-*gfp* were grown overnight in the presence of 1 mM sodium salicylate (inducer). Cell suspensions were boiled and loaded into a polyacrylamide gel. Gel bands from 50 – 75 kDa (expected size of the truncated proteins ~59 kDa) were excised and analyzed by mass spectrometry. Peptide sequences for FtsI_Trunc_ or FtsI*_Trunc_ covering 29 and 57 %, respectively, of the full length FtsI protein were obtained. Yellow highlighted sequences correspond to observed peptides. Red font corresponds to the active site mutation in FtsI* (Ser307Ala). FtsI fragments were not observed in the excised gel band from the GFP-expressing control. Further, none of the fragments from the cytoplasmic or transmembrane domain of FtsI were detected in any sample. (TIF 275 kb)

Additional file 14: **Figure S12.** Persister levels following expression of FtsI, FtsI*, FtsI_Trunc_, or FtsI*_Trunc_ in stationary phase. Cultures of MG1655 carrying pKG110-*fts*I, pKG110-ftsI*, pKG110-*ftsI*_Trunc_, or pKG110-ftsI*_Trunc_, were treated with 100 µM sodium salicylate (inducer) at t = 3 h. Cells in control culture were treated with an equal volume of water (-). At 24 h, cell cultures were washed to remove the inducer and diluted in fresh LB containing 200 µg/mL ampicillin (AMP), 5 µg/mL ofloxacin (OFL), or water (-). Survival fractions are shown for no induction controls (dashed lines) treated with ampicillin (A and E) or ofloxacin (B and F). CFU levels are shown for non-induced (dashed lines) and induced cultures (continuous lines) of MG1655 carrying pKG110-*ftsI* (C), pKG110-ftsI* (D), pKG110-*ftsI*_Trunc_ (G), or pKG110-_ftsI*__Trunc_ (H), treated with ampicillin or ofloxacin. Data represent three or more biological replicates. Each data point was denoted as mean ± s.e. (TIF 497 kb)

Additional file 15: **Figure S13.** Measurements of respiration, RNA integrity, and protein degradation following piperacillin treatment. (A) Cell cultures were treated with 200 µg/mL piperacillin (PIP-treated) at t= 4h. Cells in control groups were treated with equal volume of water (untreated). Oxygen levels and OD_600_ measurements were performed at indicated time points. (B) Cell cultures were treated with piperacillin or water at t=4 h. At t=24 h, cells were pelleted for RNA extraction. RNA integrity was determined with a Bioanalyzer using an RNA 6000 Nano Kit. For control, an early stationary phase culture (t = 6 h) was used. (C) rRNA degradation was determined based on RNA integrity values ranging from 1-10, where high values signify less degraded RNA. (D) Cultures of MO-cured carrying pQE-80L*gfp-ssrA* were grown in the presence of the inducer for *gfp-ssrA* and *mCherry* for up to 4 h. At t = 4 h, the inducer was removed and piperacillin added. GFP and mCherry fluorescence were measured immediately after addition of piperacillin (t = 4 h) and at t = 5, 6 and 7 h. Green fluorescence was normalized to red fluorescence as described in Materials and Methods. Data represent three or more biological replicates. Each data point was denoted as mean ± s.e.. * *p*<0.05 (t-test). (TIF 131 kb)

Additional file 16: **Table S1.** Bacterial strains and plasmids. (DOCX 24 kb)

Additional file 17: **Table S2.** DNA oligonucleotides. (DOCX 17 kb)

Additional file 18: **Figure S14.** Experimental workflows.Overnight cultures were diluted (1000-fold) in 2 mL of fresh LB medium in a test tube and incubated at 37 ºC with shaking (250 rpm) for 24 h. As specified in the main text, treatments were carried out at t = 4, 5 or 6 h with piperacillin (PIP), mecillinam (MEC), fosfomycin (FOS), D-cycloserine (DCS) or ampicillin (AMP) at 200 µg/mL unless otherwise noted. For overexpression of FtsI, FtsI*, FtsI_Trunc_, and FtsI*_Trunc_, sodium salicylate was added at t = 3 h. Microscopy imaging, ATP, RNA, Protein, Bocillin-FL binding, and DNA measurements were conducted on the 24 h cultures. After washing and resuspension in fresh media with or without β-lactams (AMP, CAR) and fluoroquinolones (OFL, CIP), persister, cell division, protein synthesis, PBP labeling, and plasmid DNA supercoiling assays were performed. Colony counting from persister assays were performed at 16 and 48 hr. (TIF 142 kb)

Additional file 19: **Figure S15.** Minimum inhibitory concentrations. MICs were determined by the microdilution method. Optical density (OD_600_) measurements were carried out after incubation for 16-18 h with piperacillin (PIP), ampicillin (AMP), D-cycloserine (DCS), fosfomycin (FOS), mecillinam (MEC), carbenicillin (CAR), novobiocin (NVB), ofloxacin (OFL), ciprofloxacin (CIP) or, chloramphenicol (CAM). MICs are indicated (arrow head). For a definition of the MIC, see Supplemental Materials and Methods. (TIF 204 kb)

## Data Availability

The datasets used and/or analyzed during the current study are available from the corresponding author on reasonable request.

## Abbreviations

AMP: Ampicillin
CAM: Chloramphenicol
CAR: Carbenicillin
CIP: Ciprofloxacin
CYC: D-cycloserine
DIC: Differential interference contrast
DMSO: Dimethyl sulfoxide
FOS: Fosfomycin
GFP: Green fluorescent protein
Glc: Glucose
IPTG: Isopropyl β-D-1-thiogalactopyranoside
KCN: Potassium cyanide
MEC: Mecillinam
MIC: Minimum inhibitory concentration
NVB: Novobiocin
OFL: Ofloxacin
PBP: Penicillin binding protein
PBP3: Penicillin binding protein 3
PBS: Phosphate-buffered saline
PFA: Paraformaldehyde
PIP: Piperacillin
