# Supplementary figures and images for: Stationary phase persister formation in Escherichia coli can be suppressed by piperacillin and PBP3 inhibition
Source: BMC Microbiol. 2019 Jun 24;19:140. doi: 10.1186/s12866-019-1506-7 (PMC6591824; doi:10.1186/s12866-019-1506-7)

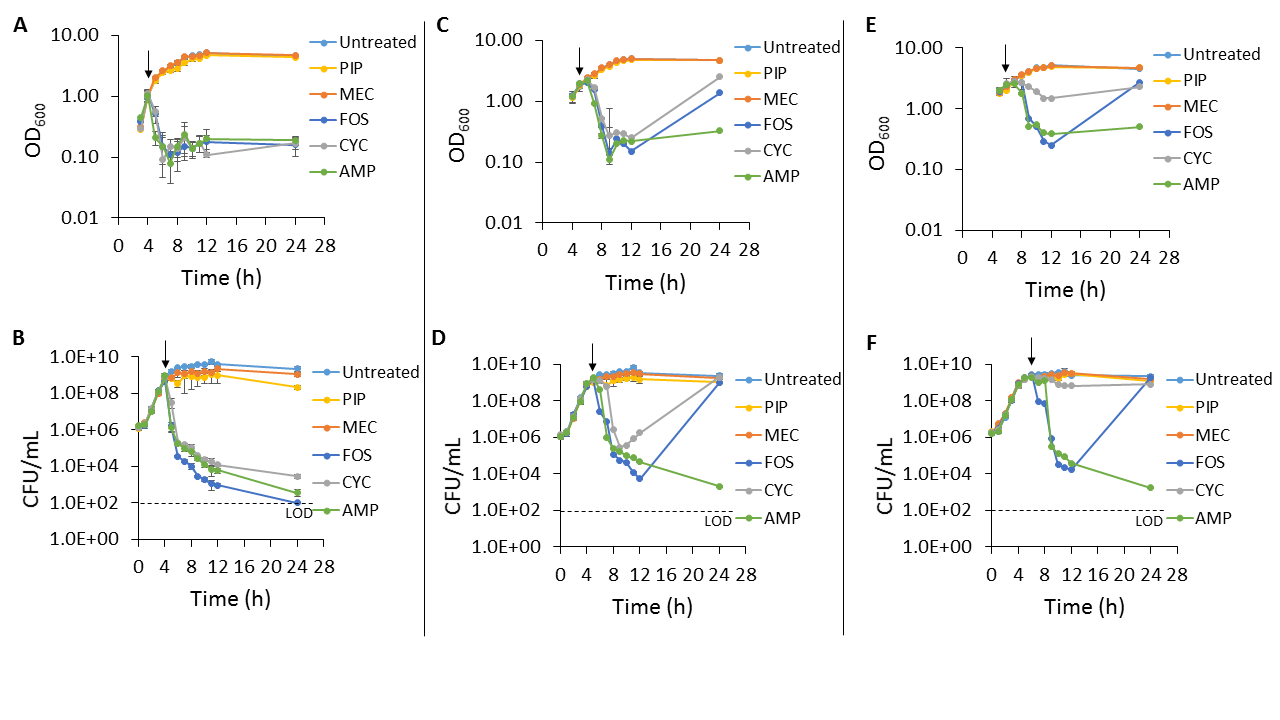

Supplement: Supplementary file 1 — Figure S1. Cell wall integrity and culturability following treatment with cell wall inhibitors. Cell cultures were treated with 200 µg/mL of piperacillin (PIP) , mecillinam (MEC), fosfomycin (FOS), D-cycloserine (CYS) or ampicillin (AMP) at t= 4 h (A and B), 5 h (C and D) or 6 h (E and F), as indicated by the arrows in each plot. Cells in control culture were treated with an equal volume of solvent, which with these antibiotics was water (untreated). OD600 (A, C and E) and CFU per mL (B, D and F) were monitored at the indicated time points. LOD: Limit of detection of the assay. Data represent at least three biological replicates. Each data point was denoted as mean ± s.e. (TIF 216 kb) [file 12866_2019_1506_MOESM1_ESM.tif]

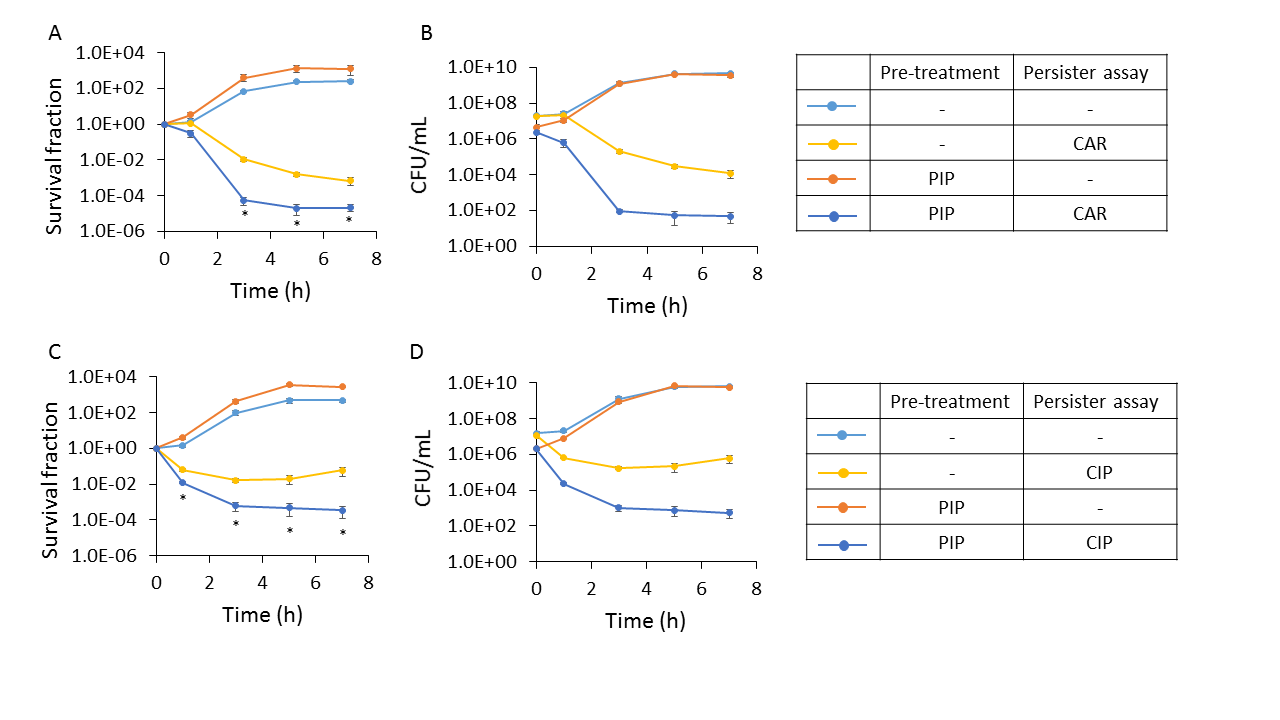

Supplement: Supplementary file 2 — Figure S2. Carbenicillin and ciprofloxacin persister assays. Cell cultures were treated with 200 µg/mL piperacillin (PIP) at t= 4 h. Cells in control culture were treated with an equal volume of water (-). At 24 h, cultures were washed to remove chemicals and diluted in fresh LB containing 200 µg/mL carbenicillin (CAR) or 1 µg/mL ciprofloxacin (CIP). Survival fractions (A and C) were monitored at the indicated time points. CFU/mL are provided (B and D). * p< 0.05 (t- test). Data represent at least three biological replicates. Each data point was denoted as mean ± s.e. (TIF 165 kb) [file 12866_2019_1506_MOESM2_ESM.tif]

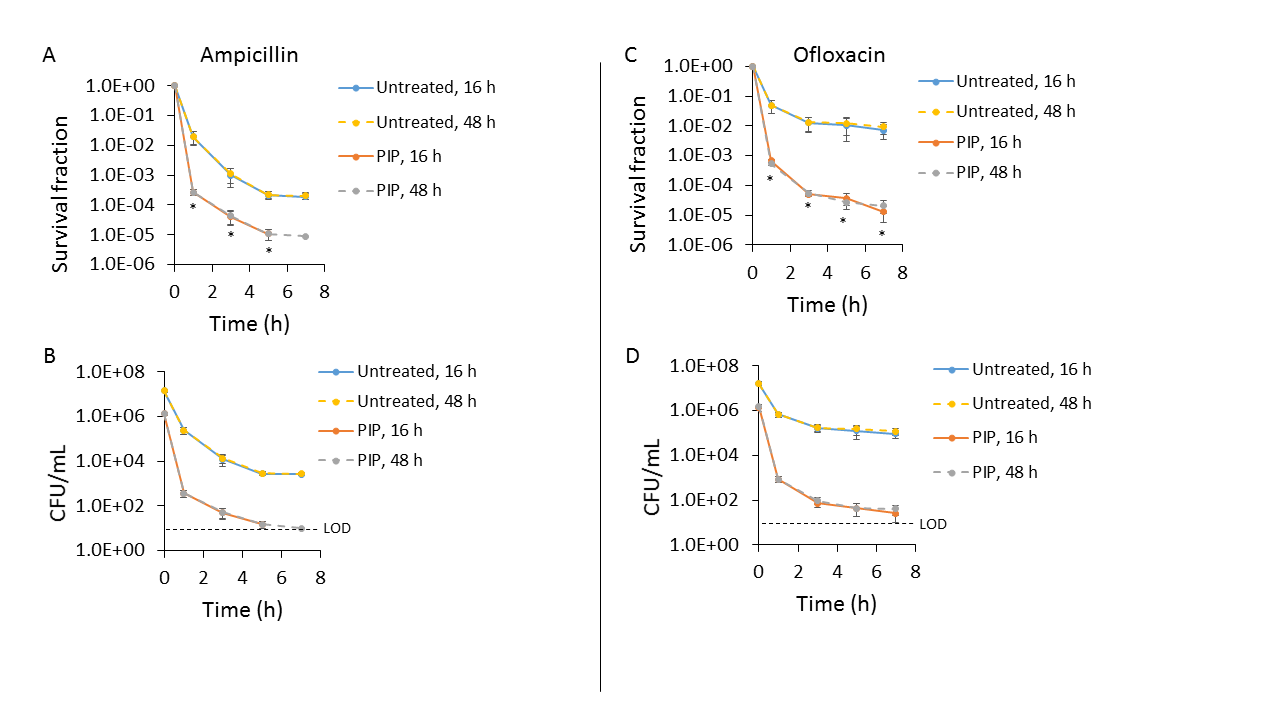

Supplement: Supplementary file 3 — Figure S3. Longer incubation of agar plates following persister assays. Persister assays were carried out as described in Figure 1. Colonies were counted after 16 and 48 h of incubation of agar plates at 37 °C. Survival fractions (A and C) and CFU/mL (B and D) are provided. LOD: Limit of detection of the assay. * p<0.05 (t-test). Data represent at least three biological replicates. Each data point was denoted as mean ± s.e. (TIF 165 kb) [file 12866_2019_1506_MOESM3_ESM.tif]

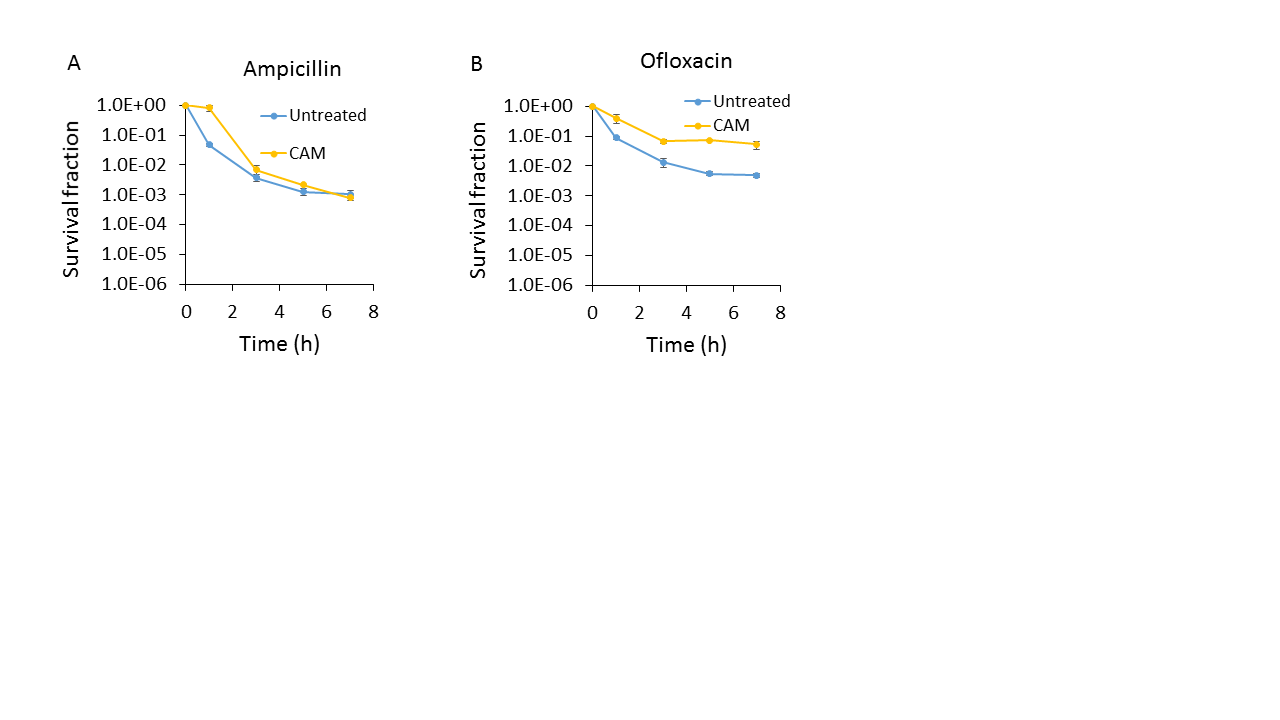

Supplement: Supplementary file 4 — Figure S4. Treatment of early stationary-phase cultures with chloramphenicol. Cultures were treated with chloramphenicol at t = 4 h. After 20 h of incubation (t = 24 h), cells were washed to remove chloramphenicol and persister assays were carried out in fresh media. Survival fractions during 7 hours of ampicillin (A) or (B) ofloxacin challenge are provided. Data represent at least three biological replicates. Each data point was denoted as mean ± s.e (TIF 104 kb) [file 12866_2019_1506_MOESM4_ESM.tif]

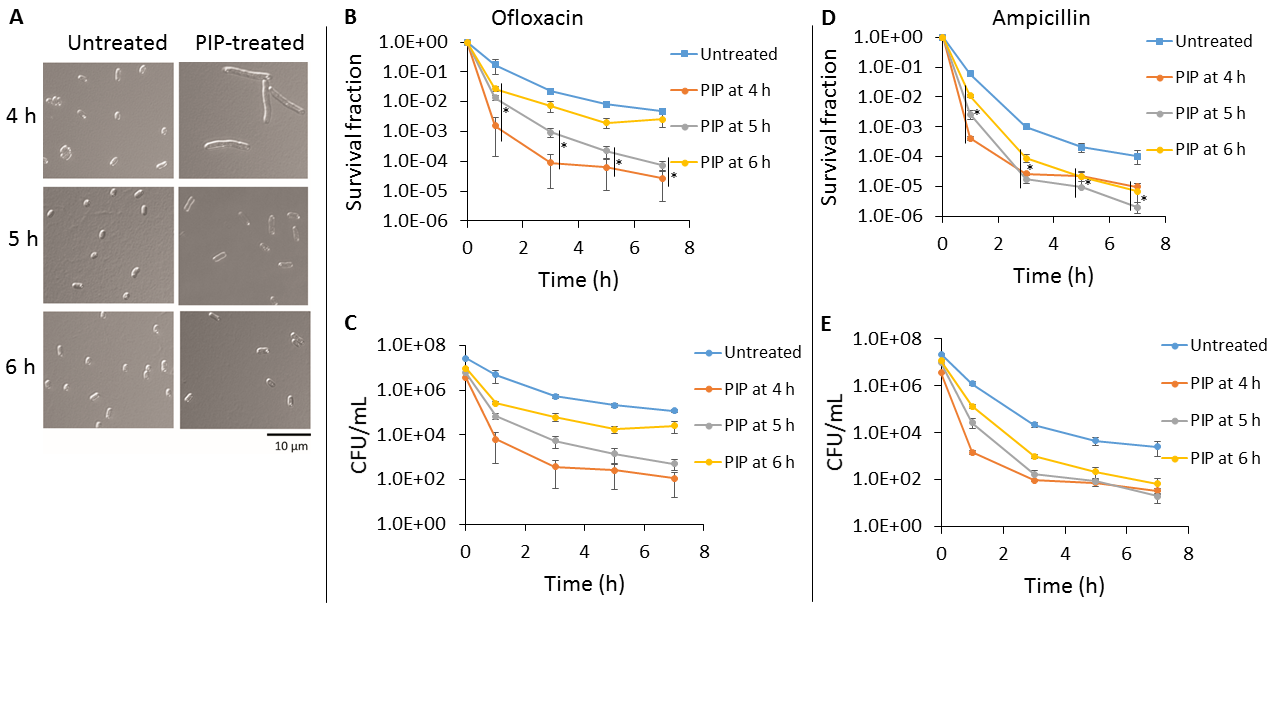

Supplement: Supplementary file 5 — Figure S5. . Piperacillin treatment during transition to stationary phase and its impact on persister formation. Cell cultures were treated with 200 µg/mL piperacillin (PIP) at t= 4, 5 or 6 h. Cells in control culture were treated with an equal volume of water (untreated). Cells were fixed at t = 24 h before washes, and imaged using a brightfield microscope (A). Cell cultures were washed to remove chemicals and diluted in fresh LB containing 200 µg/mL ampicillin or 5 µg/mL ofloxacin. CFU levels were monitored at the indicated time points. Data denoted as survival fraction (B and D) and CFU/mL (C and E). * p<0.05 (t-test). Data represent at least three biological replicates. Each data point was denoted as mean ± s.e. (TIF 342 kb) [file 12866_2019_1506_MOESM5_ESM.tif]

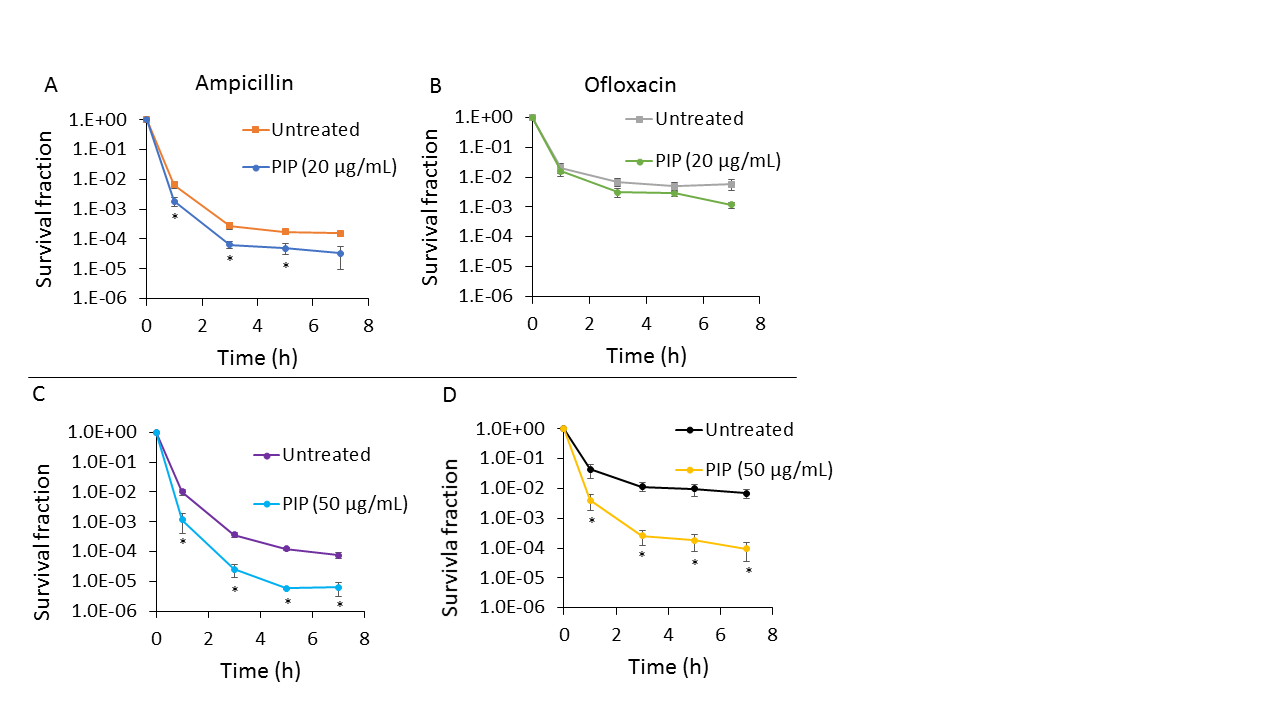

Supplement: Supplementary file 6 — Figure S6. Impact of different concentrations of piperacillin on persister levels. Cultures at early stationary phase (t = 4 h) were treated with either 20 µg/mL piperacillin (A and B) or 50 µg/mL piperacillin (C and D). Control (untreated) was treated with equal volume of water. At t = 24 h, cells were washed, diluted into fresh media and treated with either 200 µg/mL ampicillin or 5 µg/mL ofloxacin. Survival fractions were monitored for 7 h. * p<0.05 (t-test). Data represent at least three biological replicates. Each data point was denoted as mean ± s.e. (TIF 151 kb) [file 12866_2019_1506_MOESM6_ESM.tif]

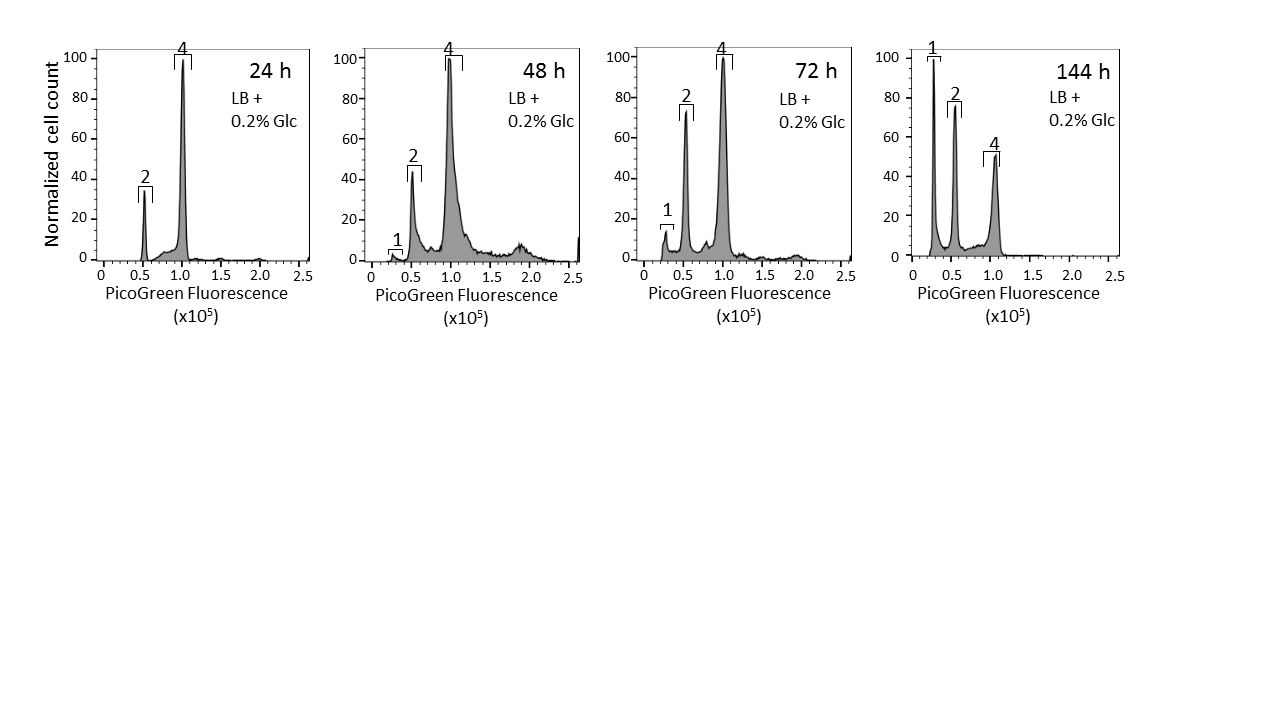

Supplement: Supplementary file 7 — Figure S7. Cultures to identify chromosome number. An overnight culture of MG1655 was diluted 107-fold into LB containing 0.2% glucose (Glc) and incubated for 144 h. One mL of sample was taken, fixed, and stained with PicoGreen at 24, 48, 72 and 144 h. Representative experiment of three biological replicates. (TIF 144 kb) [file 12866_2019_1506_MOESM7_ESM.tif]

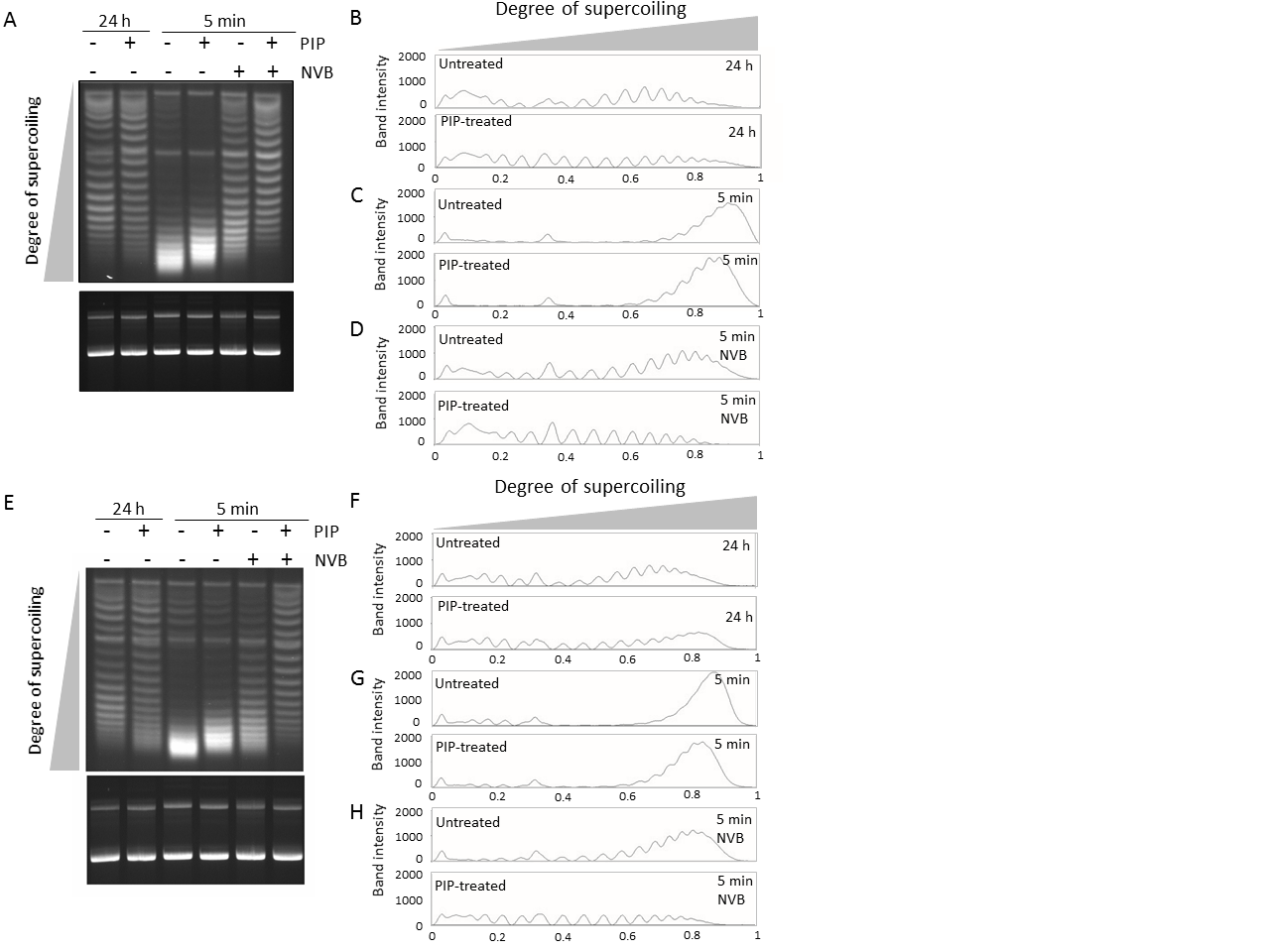

Supplement: Supplementary file 8 — Figure S8. Additional replicates of the DNA gyrase supercoiling assay during growth resumption. Cultures of MG1655-pQE-80L-kan were treated with piperacillin (PIP) or water (untreated) at t = 4.5 h (OD600 ~ 1). At t = 24 h, piperacillin (PIP) was removed by washes in fresh LB before dilution and incubation for 5 min. Where indicated novobiocin (NVB) was added to the washes and in fresh dilution media for incubation for 5 min. Plasmid DNA was extracted at t = 24 h and after 5 min incubation in fresh media. Equal amounts of plasmid DNA were loaded onto an agarose gel containing chloroquine (Top gel) (A and E) and an agarose gel without intercalator (loading control) (Bottom gel) (A and E). Chloroquine-containing gel and gel without intercalator were run for 23 h and 1 h, respectively. Densitometry scans of untreated and PIP-treated samples that were either processed at t = 24 h (B and F), washed and incubated in fresh LB for 5 min (C and G), or washed and incubated for 5 min in fresh LB in the presence of novobiocin (NVB) (D and H) prior to plasmid extraction. Gel images correspond to 2 biological repeats. (TIF 426 kb) [file 12866_2019_1506_MOESM8_ESM.tif]

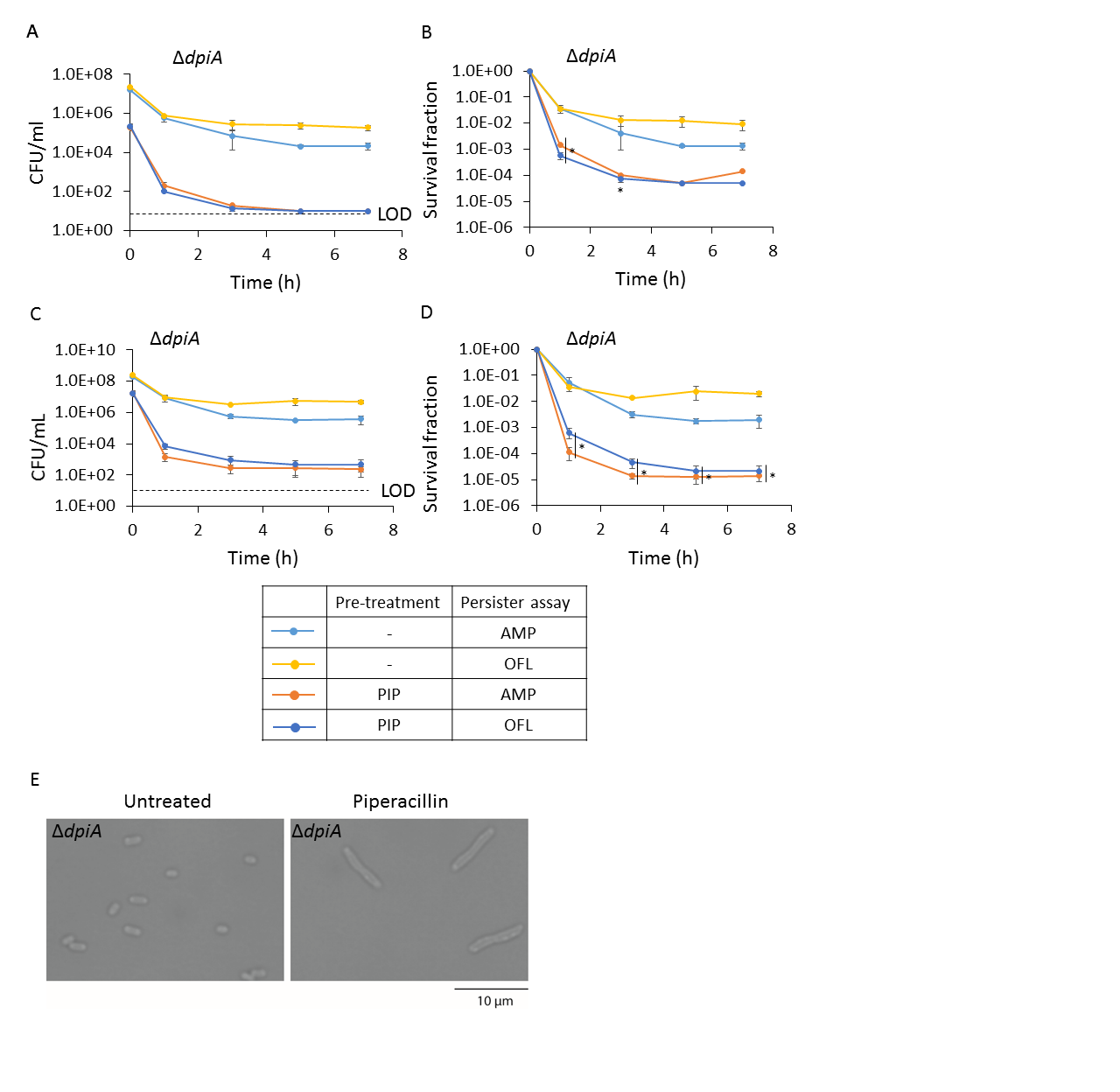

Supplement: Supplementary file 9 — Figure S9. DpiA deletion does not alter the impact of piperacillin on persister levels. MG1655ΔdpiA::kan cultures were treated with 200 µg/mL of piperacillin at t = 4 h. Control cultures were treated with an equal volume of solvent (water). At t = 24 h, cell cultures were washed to remove chemicals and diluted 100-fold in fresh LB containing 200 µg/mL ampicillin (AMP) or 5 µg/mL ofloxacin (OFL). (A) Survival fractions are shown at the indicated time points. (B) CFU levels are provided. At t = 24 h, cell cultures were washed to remove chemicals and diluted 10-fold in fresh LB containing 200 µg/mL ampicillin (AMP) or 5 µg/mL ofloxacin (OFL). (C) Survival fractions are shown at the indicated time points. (D) CFU levels are provided. (E) MG1655ΔdpiA::kan cultures were treated with piperacillin at t = 4 h. At t = 24 h, cells were fixed for microscopy analysis. * p<0.05 (t-test). Data represent three or more biological replicates. Each data point was denoted as mean ± s.e. (TIF 286 kb) [file 12866_2019_1506_MOESM9_ESM.tif]

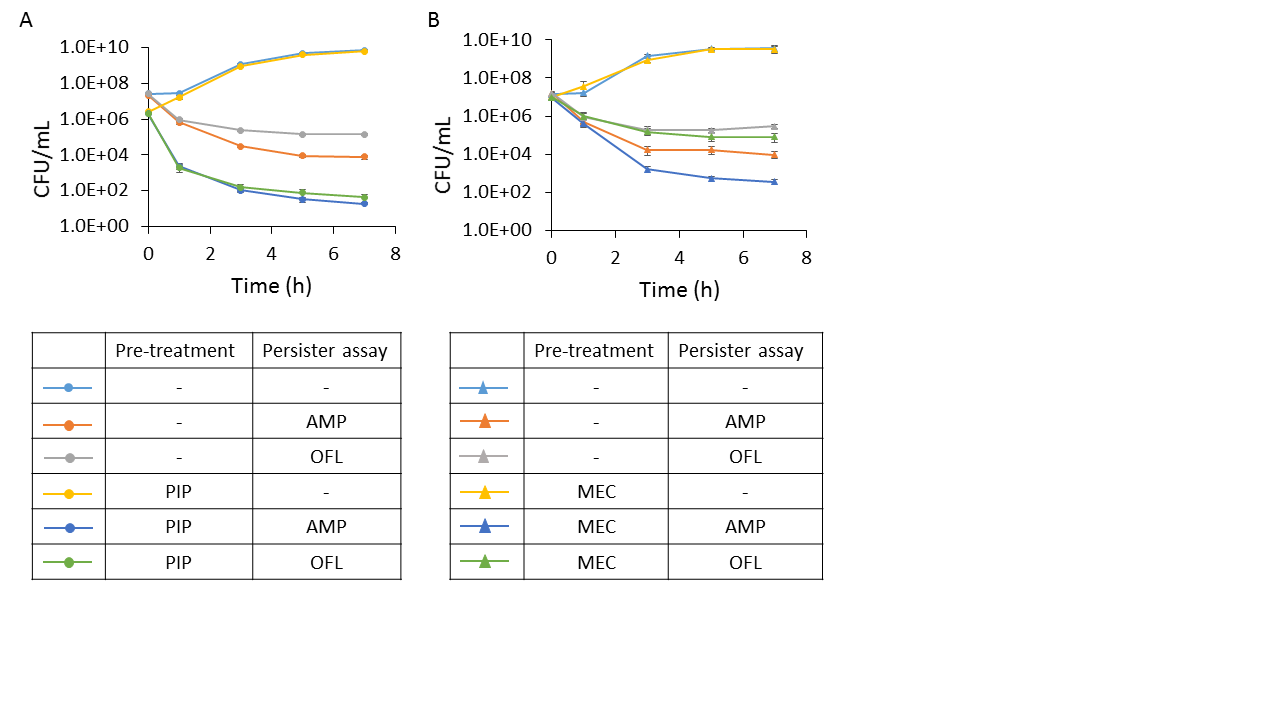

Supplement: Supplementary file 10 — Figure S19. Culturability data from stationary-phase treatment with PIP and MEC. Cell cultures were treated with 200 µg/mL piperacillin (PIP) (A) or mecillinam (MEC) (B) at t= 4 h. Cells in control culture were treated with an equal volume of solvent (water). At 24 h, cell cultures were washed to remove chemicals and diluted in fresh LB containing 200 µg/mL ampicillin (AMP), 5 µg/mL ofloxacin (OFL), or water (-). CFU levels were monitored at the indicated time points. Data represent three or more biological replicates. Each data point was denoted as mean ± s.e. (TIF 136 kb) [file 12866_2019_1506_MOESM10_ESM.tif]

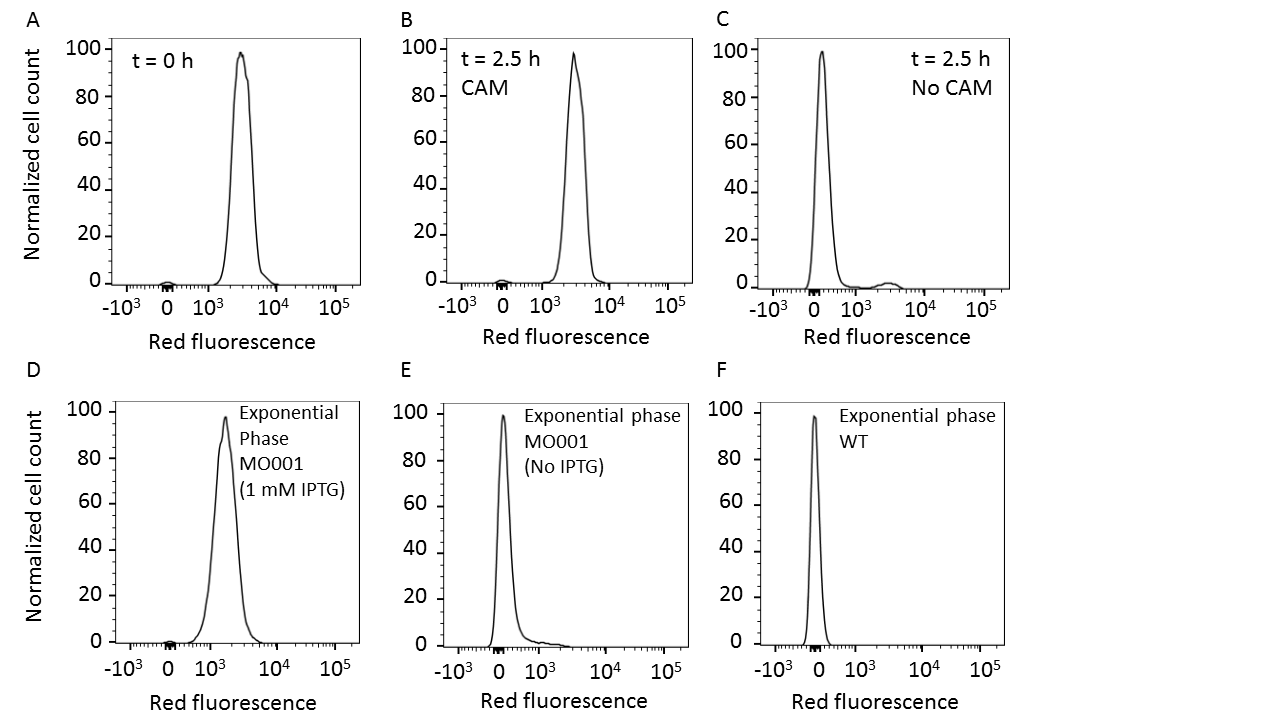

Supplement: Supplementary file 11 — Supplemental Methods. (ZIP 423 kb) [file 12866_2019_1506_MOESM11_ESM.zip › 12866_2019_1506_MOESM11_ESM/12866_2019_1506_MOESM20_ESM.tif]

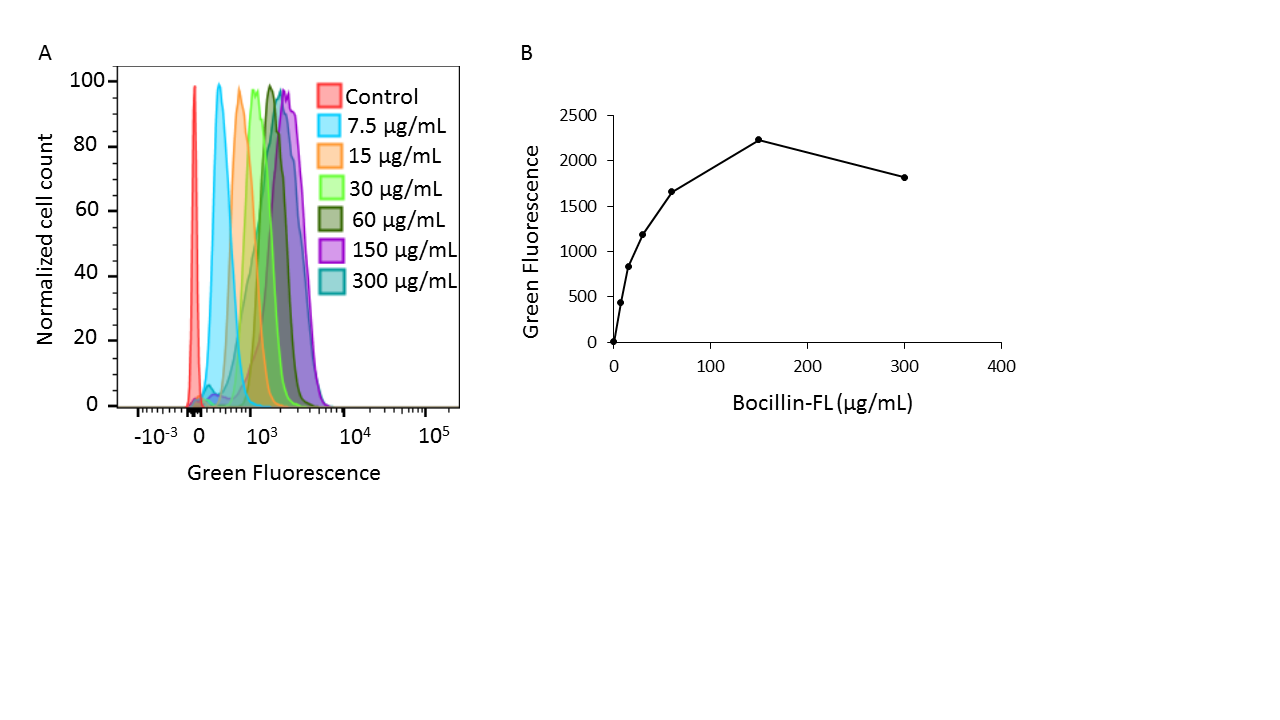

Supplement: Supplementary file 11 — Supplemental Methods. (ZIP 423 kb) [file 12866_2019_1506_MOESM11_ESM.zip › 12866_2019_1506_MOESM11_ESM/12866_2019_1506_MOESM21_ESM.tif]

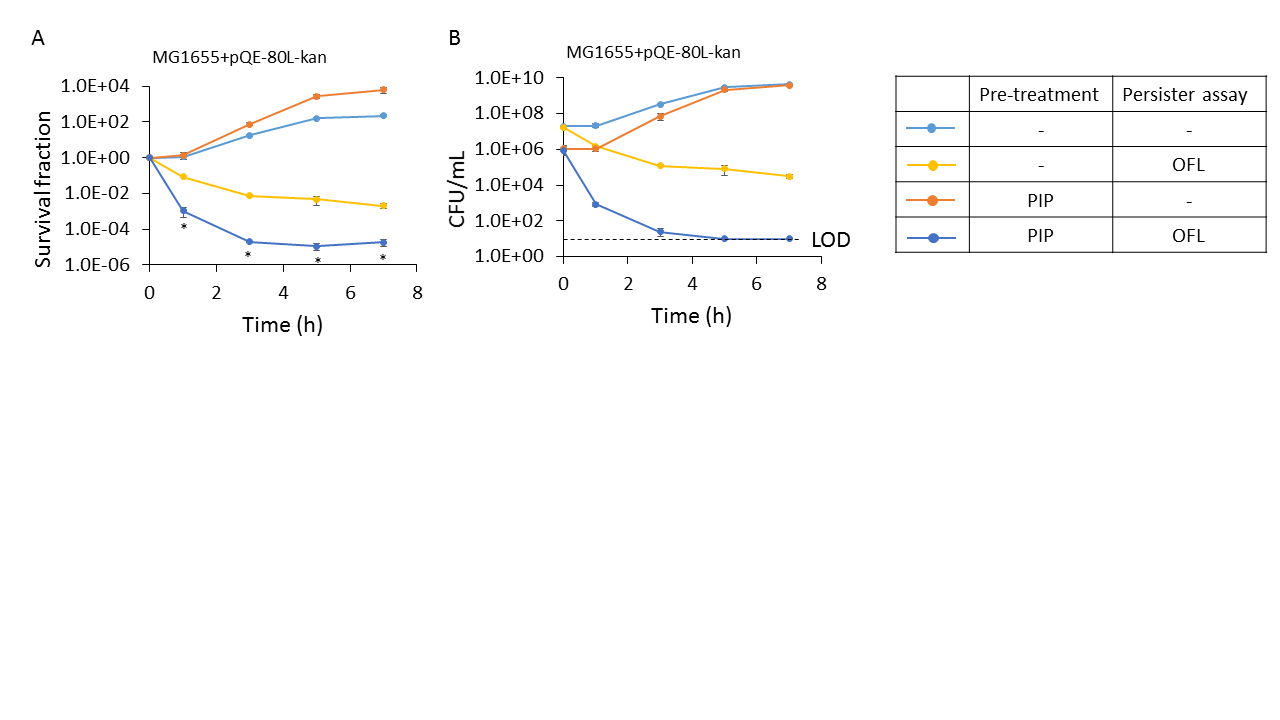

Supplement: Supplementary file 11 — Supplemental Methods. (ZIP 423 kb) [file 12866_2019_1506_MOESM11_ESM.zip › 12866_2019_1506_MOESM11_ESM/12866_2019_1506_MOESM22_ESM.tif]

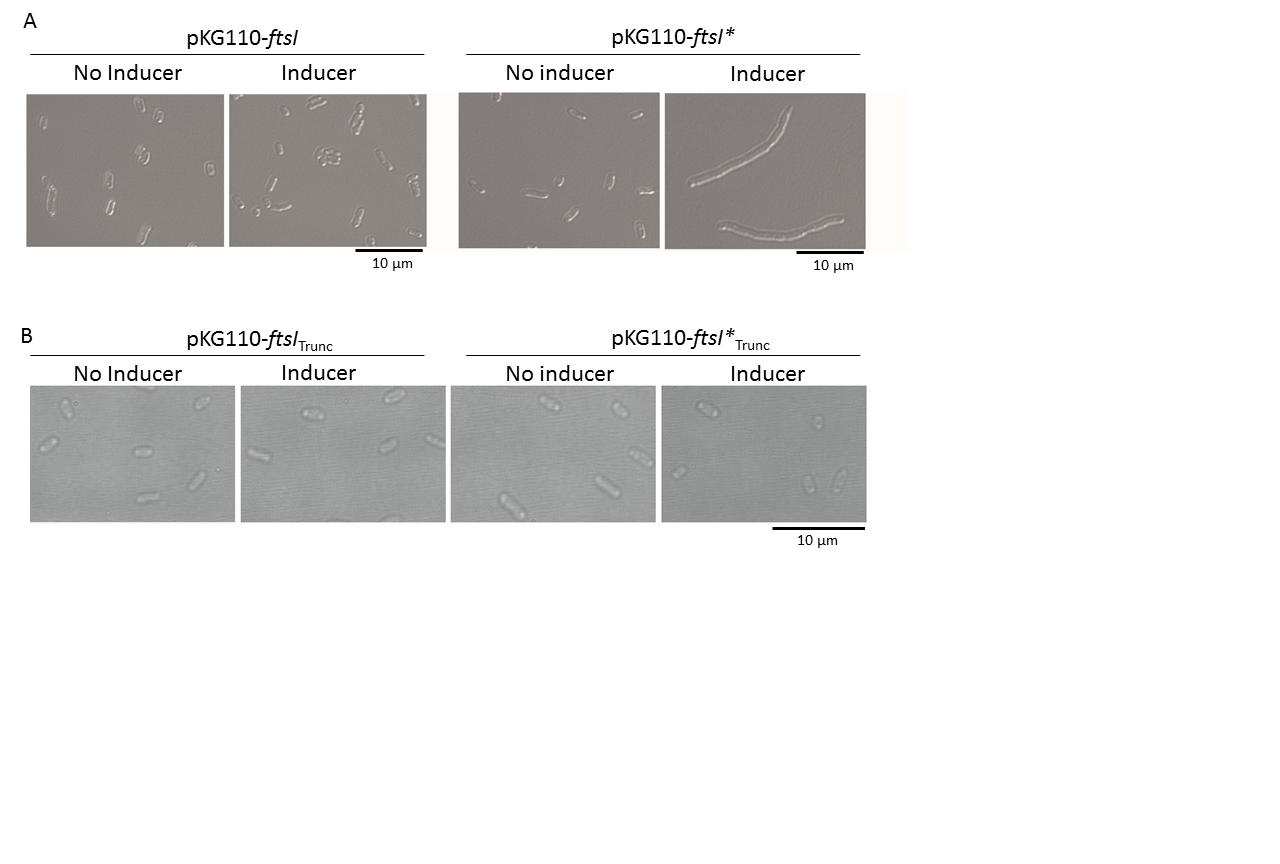

Supplement: Supplementary file 12 — Figure S10. . Expression of FtsI* resulted in filamentation whereas expression of FtsI, FtsITrunc, and FtsI*Trunc did not. Cultures of MG1655 carrying pKG110-ftsI (A, left), pKG110-ftsI* (A, right), pKG110-ftsITrunc (B, left), or pKG110-ftsI*Trunc (B, right) were grown for 24 h. At t = 3 h, sodium salicylate (100 µM) was added to induce expression of ftsI, ftsI*, ftsITrunc or ftsI*Trunc from plasmid. At 24 h incubation, cells were fixed for microscopy analysis. . (TIF 428 kb) [file 12866_2019_1506_MOESM12_ESM.tif]

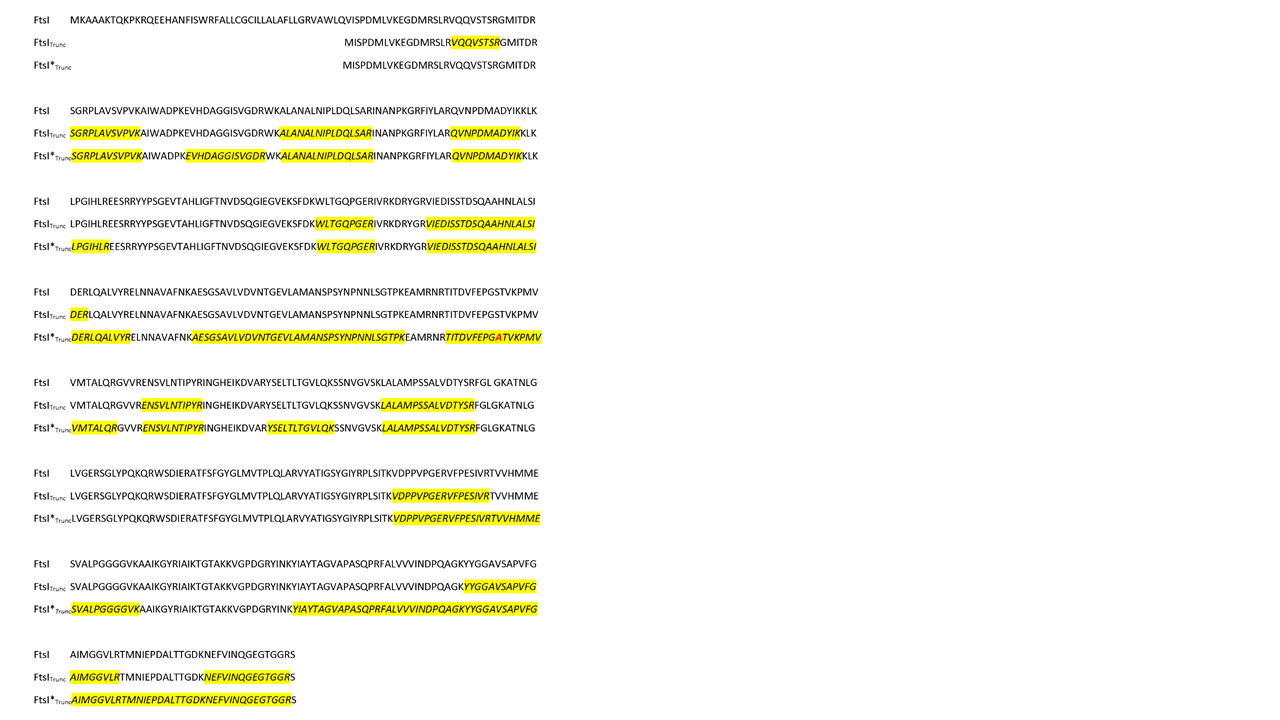

Supplement: Supplementary file 13 — Figure S11. Confirmation of FtsITrunc and FtsI*Trunc expression by mass spectrometry. Cultures of MG1655 carrying pKG110-ftsITrunc, pKG110-ftsI*Trunc, or pKG110-gfp were grown overnight in the presence of 1 mM sodium salicylate (inducer). Cell suspensions were boiled and loaded into a polyacrylamide gel. Gel bands from 50 – 75 kDa (expected size of the truncated proteins ~59 kDa) were excised and analyzed by mass spectrometry. Peptide sequences for FtsITrunc or FtsI*Trunc covering 29 and 57 %, respectively, of the full length FtsI protein were obtained. Yellow highlighted sequences correspond to observed peptides. Red font corresponds to the active site mutation in FtsI* (Ser307Ala). FtsI fragments were not observed in the excised gel band from the GFP-expressing control. Further, none of the fragments from the cytoplasmic or transmembrane domain of FtsI were detected in any sample. (TIF 275 kb) [file 12866_2019_1506_MOESM13_ESM.tif]

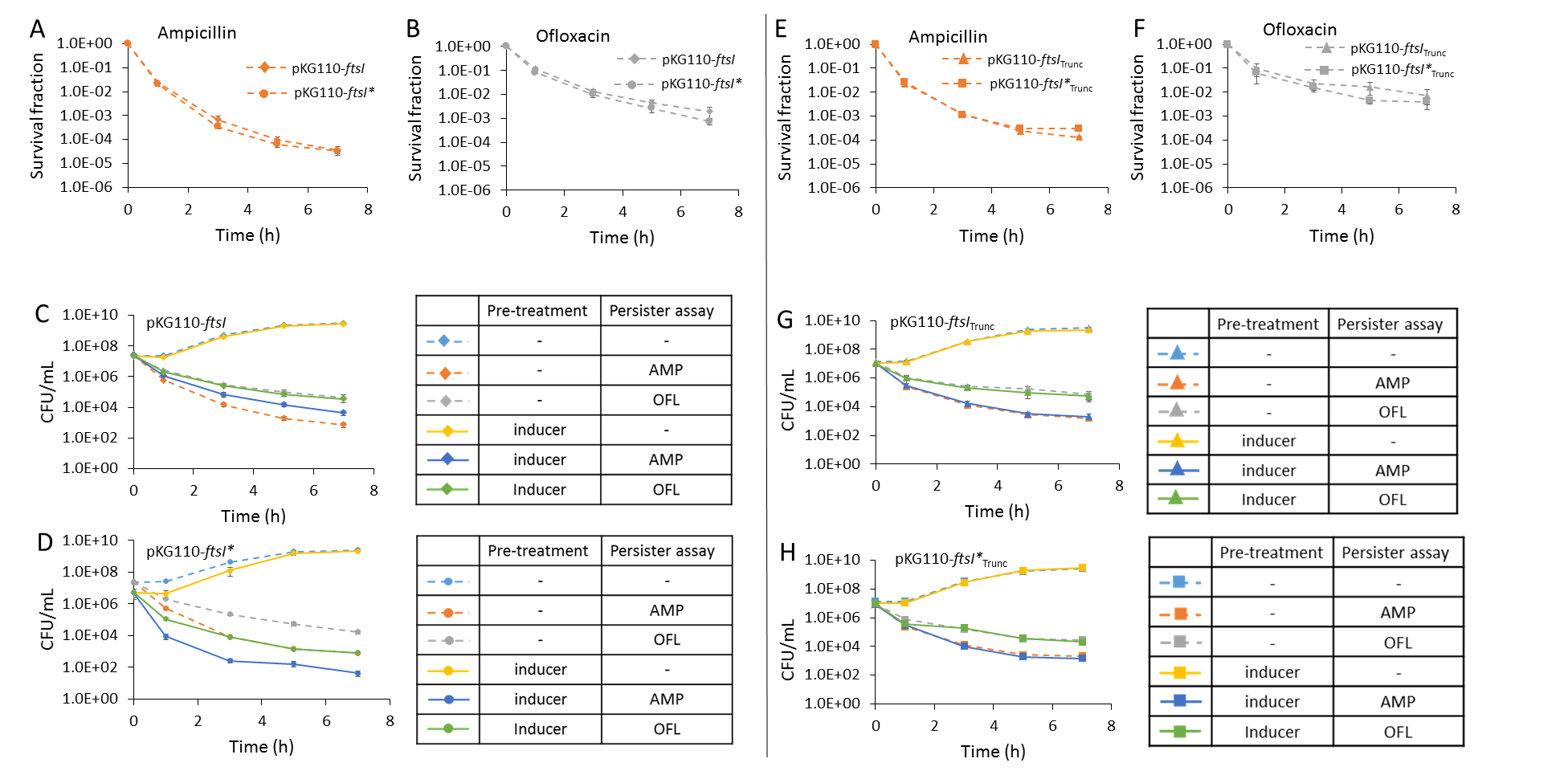

Supplement: Supplementary file 14 — Figure S12. Persister levels following expression of FtsI, FtsI*, FtsITrunc, or FtsI*Trunc in stationary phase. Cultures of MG1655 carrying pKG110-ftsI, pKG110-ftsI*, pKG110-ftsITrunc, or pKG110-ftsI*Trunc, were treated with 100 µM sodium salicylate (inducer) at t = 3 h. Cells in control culture were treated with an equal volume of water (-). At 24 h, cell cultures were washed to remove the inducer and diluted in fresh LB containing 200 µg/mL ampicillin (AMP), 5 µg/mL ofloxacin (OFL), or water (-). Survival fractions are shown for no induction controls (dashed lines) treated with ampicillin (A and E) or ofloxacin (B and F). CFU levels are shown for non-induced (dashed lines) and induced cultures (continuous lines) of MG1655 carrying pKG110-ftsI (C), pKG110-ftsI* (D), pKG110-ftsITrunc (G), or pKG110-ftsI*Trunc (H), treated with ampicillin or ofloxacin. Data represent three or more biological replicates. Each data point was denoted as mean ± s.e. (TIF 497 kb) [file 12866_2019_1506_MOESM14_ESM.tif]

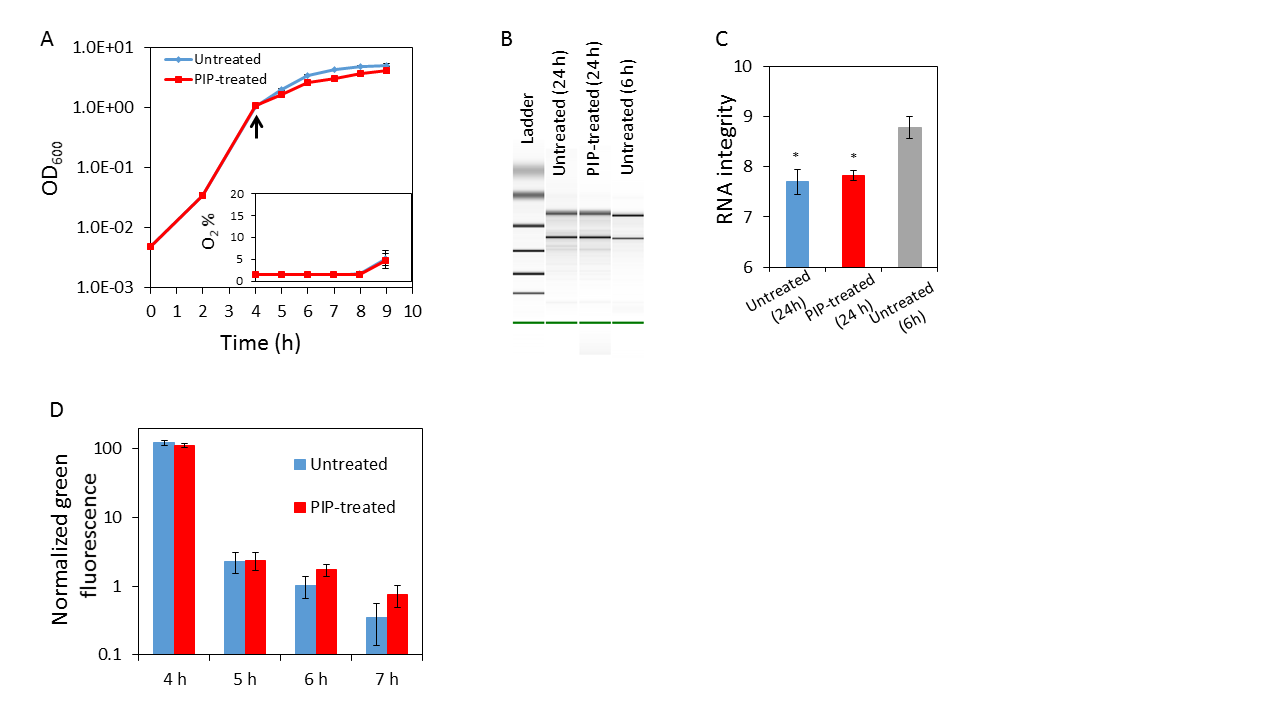

Supplement: Supplementary file 15 — Figure S13. Measurements of respiration, RNA integrity, and protein degradation following piperacillin treatment. (A) Cell cultures were treated with 200 µg/mL piperacillin (PIP-treated) at t= 4h. Cells in control groups were treated with equal volume of water (untreated). Oxygen levels and OD600 measurements were performed at indicated time points. (B) Cell cultures were treated with piperacillin or water at t=4 h. At t=24 h, cells were pelleted for RNA extraction. RNA integrity was determined with a Bioanalyzer using an RNA 6000 Nano Kit. For control, an early stationary phase culture (t = 6 h) was used. (C) rRNA degradation was determined based on RNA integrity values ranging from 1-10, where high values signify less degraded RNA. (D) Cultures of MO-cured carrying pQE-80Lgfp-ssrA were grown in the presence of the inducer for gfp-ssrA and mCherry for up to 4 h. At t = 4 h, the inducer was removed and piperacillin added. GFP and mCherry fluorescence were measured immediately after addition of piperacillin (t = 4 h) and at t = 5, 6 and 7 h. Green fluorescence was normalized to red fluorescence as described in Materials and Methods. Data represent three or more biological replicates. Each data point was denoted as mean ± s.e.. * p<0.05 (t-test). (TIF 131 kb) [file 12866_2019_1506_MOESM15_ESM.tif]

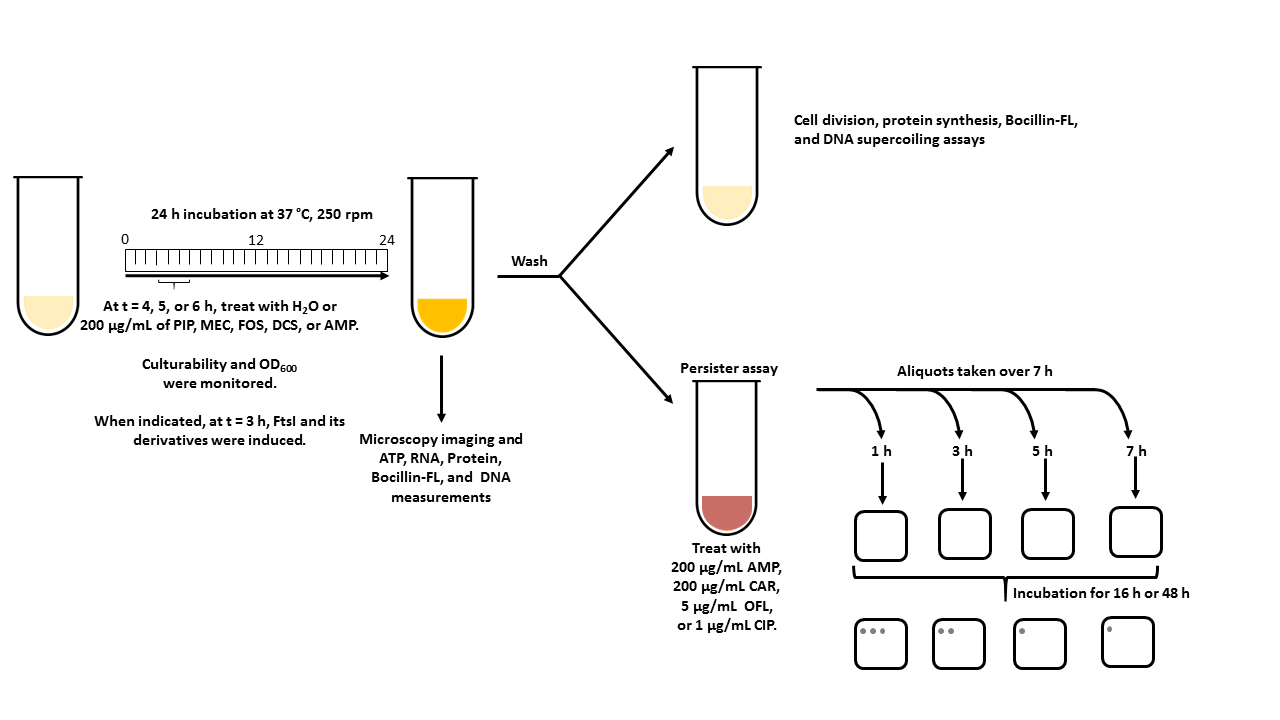

Supplement: Supplementary file 18 — Figure S14. Experimental workflows.Overnight cultures were diluted (1000-fold) in 2 mL of fresh LB medium in a test tube and incubated at 37 ºC with shaking (250 rpm) for 24 h. As specified in the main text, treatments were carried out at t = 4, 5 or 6 h with piperacillin (PIP), mecillinam (MEC), fosfomycin (FOS), D-cycloserine (DCS) or ampicillin (AMP) at 200 µg/mL unless otherwise noted. For overexpression of FtsI, FtsI*, FtsITrunc, and FtsI*Trunc, sodium salicylate was added at t = 3 h. Microscopy imaging, ATP, RNA, Protein, Bocillin-FL binding, and DNA measurements were conducted on the 24 h cultures. After washing and resuspension in fresh media with or without β-lactams (AMP, CAR) and fluoroquinolones (OFL, CIP), persister, cell division, protein synthesis, PBP labeling, and plasmid DNA supercoiling assays were performed. Colony counting from persister assays were performed at 16 and 48 hr. (TIF 142 kb) [file 12866_2019_1506_MOESM18_ESM.tif]

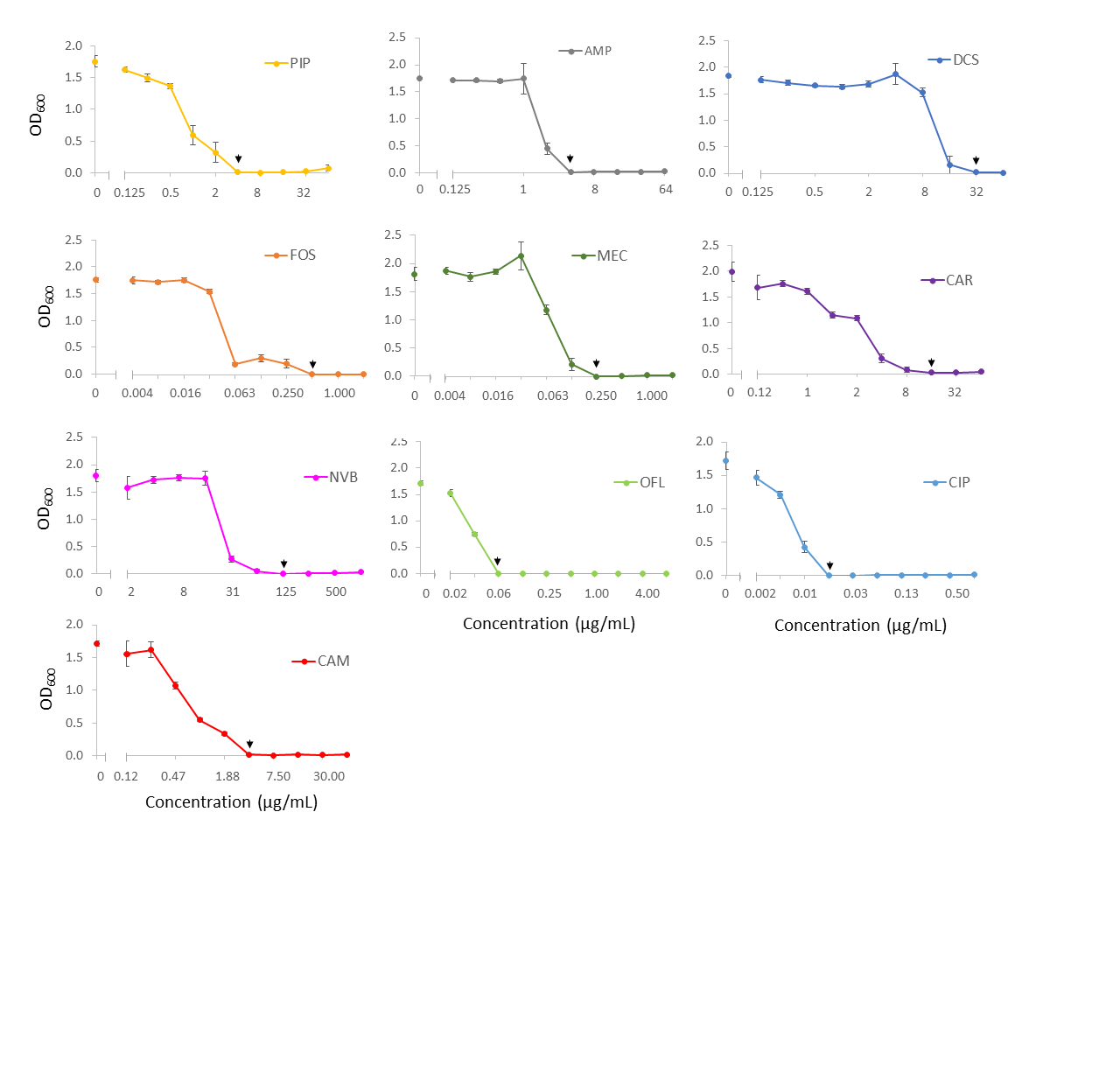

Supplement: Supplementary file 19 — Figure S15. Minimum inhibitory concentrations. MICs were determined by the microdilution method. Optical density (OD600) measurements were carried out after incubation for 16-18 h with piperacillin (PIP), ampicillin (AMP), D-cycloserine (DCS), fosfomycin (FOS), mecillinam (MEC), carbenicillin (CAR), novobiocin (NVB), ofloxacin (OFL), ciprofloxacin (CIP) or, chloramphenicol (CAM). MICs are indicated (arrow head). For a definition of the MIC, see Supplemental Materials and Methods. (TIF 204 kb) [file 12866_2019_1506_MOESM19_ESM.tif]
