# Supplementary material for: Stationary phase persister formation in Escherichia coli can be suppressed by piperacillin and PBP3 inhibition
Source: BMC Microbiol. 2019 Jun 24;19:140. doi: 10.1186/s12866-019-1506-7 (PMC6591824; doi:10.1186/s12866-019-1506-7)
Supplement: Supplementary file 11 — Supplemental Methods. (ZIP 423 kb) [file 12866_2019_1506_MOESM11_ESM.zip › 12866_2019_1506_MOESM11_ESM/12866_2019_1506_MOESM11_ESM.docx]

**Supplemental Methods**

**Bacterial strains.** MO001 was previously generated (1), and it carries a chromosomal integration of mCherry under the control of isopropyl β-D-1-thiogalactopyranoside (IPTG). MO-cured was derived from MO001 by removing the kanamycin resistance cassette with FLP recombinase (2). MG1655Δ*dpiA*::kan was generated by P1 transduction using strain JW0612 from the Keio collection as the donor strain (3) (Supplementary Table 1).

**Chemicals, media and growth conditions.** In all experiments, we used distilled water purified using a Millipore Milli-Q lab water system (Burlington, MA). Unless noted, all chemicals were purchased from Fisher Scientific or Sigma Aldrich. Piperacillin was purchased from Chem-Impex International, Inc. (Wood Dale, IL). Ofloxacin was purchased from AK Scientific, Inc. (Union City, CA). IPTG was purchased from Gold Biotechnology (St Louis, MO). Fluorescent particles for cell counts were purchased from Spherotech, Inc. (Lake Forest, IL). Quant-iT™ PicoGreen® dsDNA Reagent was supplied as a solution in dimethyl sulfoxide (DMSO) and dilutions were made in 25% DMSO or phosphate-buffered saline (PBS) as indicated. Sodium salicylate stock solution at 1 M was a gift from Zemer Gitai. All chemicals were dissolved in water except for chloramphenicol, ofloxacin, and ciprofloxacin which were dissolved in DMSO, 20 mM NaOH, and 0.02 N HCl, respectively. All chemical solutions were sterile filtered using a 0.22 µm syringe filter purchased from Merck Millipore Ltd (Burlington, MA), except for those containing DMSO. Bocillin-FL was supplied as a solution in DMSO and dilutions were made in PBS. Kanamycin and chloramphenicol were used for selection at 50 µg/mL and 20 µg/mL, respectively. Chloramphenicol was used at 100 µg/mL for inhibition of protein synthesis. D-cycloserine, mecillinam, and fosfomycin were used at 200 µg/mL. Novobiocin was used at 60 µg/mL. LB medium contained per liter 10 g of tryptone, 5 g of yeast extract, and 10 g of NaCl; LB-agar plates were made with LB medium containing 15 g/L of agar; and both were sterilized by autoclaving. Overnight cultures of MG1655 strain were prepared from a frozen 25% glycerol stock (stored at -80 °C) in 2 mL LB medium in a test tube and cultured at 37 ºC with shaking (250 rpm) for 24 h. Overnight cultures were diluted (1000-fold) in 2 mL of fresh LB medium in a test tube and incubated at 37 ºC with shaking (250 rpm) for another 24 h. During the second 24 h incubation, treatments were carried out with the different chemicals at the indicated concentrations and times during transition from exponential to stationary phase (Supplementary Figure 14).

**Plasmid constructions.** Plasmid pWM2765 was a gift from Zemer Gitai. This plasmid was used to clone wild type FtsI and its active site mutant FtsI* (Ser307Ala). Plasmid pWM2765 (4) is a derivative of pKG110 (5) which carries a sodium salicylate inducible promoter, P_G_, for expression of *nahG*, a regulatory gene, *nahR,* under the control of P_R_ from *Pseudomonas putida* G7 (ATCC 17485), and a chloramphenicol resistance marker. pWM2765 was digested with MfeI and XhoI. Double digestion with MfeI and XhoI excises part of the regulatory gene sequence along with the entire P_R_ and P_G_ promoter sequence. These sequences were reintroduced along with wild type *ftsI* or the active site mutant (*ftsI**) sequence. A fragment containing the *Mfe*I site attached to *nahR* sequence and the P_R_ and P_G_ promoter sequence was fused to *ftsI* or *ftsI** containing a downstream *Xho*I cut site by overlap PCR. The primers used for this construction are listed in Supplementary Table 2. The Ser307Ala mutation of *ftsI** was obtained by overlap PCR using MG1655 genomic DNA as template. The fused PCR product obtained following overlap PCR was cut with MfeI and XhoI and cloned into the corresponding sites in pWM2765. Truncated *ftsI* (*ftsI*_Trunc_) and *ftsI** (*ftsI**_Trunc_) encoding for residues 1 followed by 48-577, and lacking the cytoplasmic and transmembrane domains (6) were constructed using Gibson Assembly Cloning Kit (Qiagen) following the manufacturer’s instructions. PCR primers with overlapping sequences were designed using NEBuilder tool and DNA sequences are provided in Supplementary Table 2. Plasmid pKG110 carrying *ftsI* or *ftsI** was used as template for PCR amplification of truncated FtsI and FtsI*, respectively. The start codon of *ftsI* was included in the primer sequence followed by codon encoding residue 48. Primers used for confirmation of constructs by Sanger sequencing are listed in Supplementary Table 2. For overexpression of GFP and *ssrA*-tagged GFP, plasmids pQE-80L-*gfp* and pQE-80L*gfp-ssrA* were used, respectively. These plasmids were previously constructed using a plasmid pQE-80L (Qiagen, Valencia, CA) variant containing synthetic *T5* promoter, *lacI* expression from the *lacI^q^* promoter, and a kanamycin resistance marker (2).

**Determination of minimum inhibitory concentrations (MICs).** Protocols for determination of MICs using the microdilution method were carried out as described (7). Optical density (OD_600_) was measured using a plate reader after 16-18 h incubation at 37 °C with the antibiotics. The minimum inhibitory concentration was defined as the lowest concentration at which no significant growth was measured.

**Impact of cell wall inhibitors on stationary-phase culturability.** The impact of cell wall inhibitors on stationary phase culturability and cell wall integrity was determined by enumerating CFUs and monitoring OD_600_ in cultures treated with a cell wall inhibitor at the transition from exponential to stationary phase. See Supplementary Figure 14 for an illustration of the treatment protocol. Control cultures were treated with equal volumes of solvent. CFUs were enumerated and OD_600_ measured every hour for the first 12 hours and after 24 h. For CFU determination, 100 µL samples were washed with 900 µL PBS, cells pelleted by centrifugation at 21,130 g for 3 minutes, and 900 µL of the supernatant was removed. This washing procedure was repeated one more time. After these washes the concentrations of most antibiotics used were below their respective MICs (Supplementary Figure 15) (7). Ten microliters of washed cells were serially diluted in 90 µL PBS. Ten microliters of each dilution were spotted on LB agar and incubated for 16 h at 37 °C. Spots that were counted all came from dilutions where the antibiotic concentration was below its MIC.

**Persister assay.** Persister levels were determined by enumerating CFUs in cultures treated with ampicillin (200 μg/mL = 50-fold MIC), carbenicillin (200 μg/mL = 12.5-fold MIC), ofloxacin (5 μg/mL = 83.3-fold MIC), or ciprofloxacin (1 μg/mL = 66.6-fold MIC) over a course of 7 hours. To remove chemicals that had been added to the cultures prior to the persister assay, cell cultures were washed twice with fresh LB by centrifugation at 21,130 g for 3 minutes, followed by removal of supernatant, and cell resuspension in 2 mL LB. Washed cultures were diluted (100-fold) in 1 mL of LB, except for strain Δ*dpiA* which was diluted 10-fold. At 100-fold dilutions, the culturability of Δ*dpiA* treated with piperacillin throughout stationary phase was too low to observe CFUs at some time points (Supplementary Figure 9), which necessitated use of the 10-fold diluted inoculum. Ten microliter samples were taken before addition of ampicillin, carbenicillin, ofloxacin, or ciprofloxacin for determination of CFUs prior to treatment (t = 0 h). Following the addition of ampicillin, carbenicillin, ofloxacin, or ciprofloxacin, 100 µL samples were taken at 1, 3, 5 and 7 h. At t = 0 h, cells were washed with PBS once, followed by resuspension in 100 µl PBS. At the post-treatment time point, cells were washed with PBS twice, in order to achieve antibiotic concentrations below the respective MICs, followed by resuspension in 100 µL of PBS. Ten microliters were used for serial dilutions in 90 µL of PBS. Ten microliters per dilution were spotted onto LB agar. LB agar plates were incubated at 37 °C for 16 h unless noted otherwise.

**Chromosome staining with PicoGreen reagent.** Staining of DNA content with PicoGreen was carried out following a previously described protocol (8). One milliliter of a stationary-phase culture (t = 24 h) previously treated with piperacillin at t = 4 h was transferred to 9 mL of 70% ethanol and stored at 4 °C overnight. Untreated cultures were used as control. Following overnight incubation, ethanol was removed by centrifugation, pellets were air-dried overnight at room temperature, and cells were resuspended in 1 mL of PBS. For staining, cells were diluted to an OD_600_ ~ 0.4 in PBS. One-hundred microliters of PicoGreen reagent (PicoGreen stock diluted 100-fold in 25% DMSO) was added to 500 µL of cell suspension and incubated in the dark at room temperature for 3 h. Stained cells were further stained with 1 mL of PicoGreen reagent diluted 1000-fold in PBS prior to analysis by flow cytometry. For unstained control cells, 100 µL of a 25% DMSO solution was added to 500 µL of cell suspension (OD_600_ ~ 0.4) followed by incubation for 3 h at room temperature. Before flow cytometry analysis, 1 mL of DMSO diluted 1000-fold in PBS was added to the unstained control cells. Unstained control cells were used to set a gate for PicoGreen-negative population. Controls for chromosome number were prepared as described previously (9). Briefly, an overnight culture of MG1655 was 10^7^-fold diluted and grown in 25 ml of LB containing 0.2% glucose in baffled 250 mL flasks at 37 °C with shaking (250 rpm). One-milliliter samples were taken, fixed, and stained, as described above, at 24, 48, 72 and 144 h. Samples from time points 24, 48 and 72 h were stored at 4 °C in ethanol until sample collected at 144 h was ready to be processed for PicoGreen staining. A representative experiment carried out with controls for chromosome number is shown (Supplementary Figure 7). We observed that a single chromosome corresponds to 0.2-0.35x10^5^ fluorescence units, two chromosomes correspond to 0.45-0.6x10^5^ fluorescence units, four chromosomes correspond to 0.9-1.1x10^5^ fluorescence units, and eight chromosomes correspond to 1.8-2.2x10^5^ fluorescence units. We used an LSRII flow cytometer (BD Biosciences, San Jose, CA). Cell populations were identified with forward and side scatter parameters. The laser emission was set at 488 nm and fluorescence was collected using a green fluorescene filter (525/50 nm-band-pass filter). Data were acquired and analyzed using the FACSDiVa software and FlowJo, respectively.

**ATP measurements.** ATP content was measured using BacTiter-Glo Microbial Cell Viability Assay (Promega) following the manufacturer’s instructions. Cells treated with piperacillin at t = 4 h and controls (water-treated) were diluted at t = 24 h to OD_600_ ~ 0.1 in spent media and 100 µL was mixed with 100 µL of Bactiter-Glo reagent. A standard curve with known ATP concentrations (0, 0.125, 0.25, 0.5 and 1 µM) was prepared for both untreated and piperacillin-treated cultures using an ATP stock solution prepared in spent media from untreated and piperacillin-treated cultures, respectively. ATP concentrations were divided by the cell counts obtained by flow cytometry (protocol for cell counts described below).

**Protein content determination by the Bradford method.** Protein concentration was measured as described previously (2). One milliliter of a stationary-phase culture (t = 24 h) previously treated with piperacillin or water at t = 4 h was washed and suspended in PBS. Cells were lysed by sonication for 20 min at 10% amplitude on ice (Fisher model CC-18 sonicator). CFUs were determined before and after sonication to ensure a ~ 99.9% lysis of the sample. Lysates were centrifuged for 3 min (21,130 g) at 4 °C to pellet insoluble material. Five microliters of soluble lysate was mixed with 250 µL of Bradford buffer in a 96-well flat bottom plate. Samples were incubated for 15 min at room temperature in the dark. Absorbance at 595 nm was measured using a plate reader (Synergy H1 Hybrid Reader, BioTek). A standard curve was prepared with bovine serum albumin solutions (0, 0.125, 0.25, 0.5, and 1 mg/mL). Protein content was calculated as [mg of protein/cell] by dividing the milligrams of protein in 1 mL by the number of cells in 1 mL. The cell counts were enumerated in the test samples by flow cytometry using counting beads as described below.

**RNA isolation.** Total RNA was purified with RNeasy extraction kit using the manufacturer’s protocol (Qiagen). Briefly, ~10^8^-10^9^ cells were mixed with RNAprotect, lysed with lysozyme, and treated with Proteinase K. For RNA integrity quantification, cells were further mechanically disrupted with acid-washed glass beads (Sigma). On-column DNA digestion with DNase I (Qiagen) was carried out to remove DNA from samples. For RNA integrity quantification, isolated total RNA samples were analyzed with a bioanalyzer using an RNA 6000 Nano kit (Agilent Technologies, Inc, Santa Clara, CA) as described previously (2). For RNA content per cell [mg of RNA/cell], the milligrams of RNA (determined by spectrophotometric analysis using a plate reader) in 1 mL was divided by the number of cells in 1 mL, as determined by flow cytometry with counting beads (procedure described below).

**Enumeration of cell counts by flow cytometry.** Ten microliters of stationary-phase cultures were collected and transferred to 90 µL of cold 70% ethanol followed by overnight incubation at 4 °C. Ethanol was removed by centrifugation and pellets were air-dried overnight followed by suspension of cells in 500 µL of PBS. Four-hundred and fifty microliters of cell suspension were transferred to 5 mL round-bottom polystyrene tubes followed by the addition of 50 µL of fluorescent particles with a known concentration (1x10^6^ fluorescent particles per mL). Enumeration of cell counts was carried out using an LSRII flow cytometer (BD Biosciences, San Jose, CA). Calculations for determination of the number of cells per µL was carried out as indicated by the manufacturer (Spherotech, Inc.). Cell populations and fluorescent particles were identified with forward and side scatter parameters. The samples were excited at 420 nm and fluorescence was collected using a green fluorescene filter (525/50 nm-band-pass filter). Data were acquired using the FACSDiVa.

**Cell division assay.** Overnight cultures of MO001 cells (*E. coli* with *mCherry* expression cassette) were diluted 1000-fold in 2 mL of LB medium containing 1 mM IPTG, and incubated. At 4 h, cultures were treated with piperacillin (200 µg/mL). Control cultures were treated with equal volumes of solvent (water). At t=24 h, cells cultures were washed with LB twice by centrifugation at 21,130 g for 3 minutes. Washed cells were resuspended in 2 mL of LB and suspensions were diluted 100-fold in 1mL of LB without IPTG and incubated for 2.5 h at 37 ºC. At t=0 h and 2.5 h, cells were washed in PBS, suspended in the same buffer and analyzed with a flow cytometer to quantify mCherry levels. Cell populations were identified with forward and side scatter parameters. The gates of the growing and non-growing cell populations were determined using overnight cultures of MO001 grown in the presence of 1 mM IPTG. These overnight cultures were washed and diluted into LB without IPTG in the absence or presence of 50 µg/mL chloramphenicol (Supplementary Figure 16A-C) before incubation for 2.5 h at 37 °C. To determine background fluorescence, overnight cultures of MO001 and wild type MG1655 were used. MO001 was grown in the absence of IPTG and diluted into LB with or without 1 mM IPTG for further incubation for 2.5 h (Supplementary Figure 16D and 16E). MG1655 was diluted and incubated for 2.5 h to determine background fluorescence (Supplementary Figure 16F). We used an LSRII flow cytometer (BD Biosciences, San Jose, CA) to measure fluorescence intensities. The samples were excited at 533 nm and red fluorescence was collected using a red (610/20) bandpass filter. Data were acquired and analyzed using the FACSDiVa software and FlowJo, respectively.

**Green fluorescent protein expression assay.** Overnight cultures of MG1655 carrying pQE-80L plasmid variant with an inducible *gfp* expression cassette (2) were diluted 1000-fold in 2 mL of LB medium with 50 µg/mL kanamycin and cultured at 37 ºC with shaking. After 4 h, cultures were treated with piperacillin (200 µg/mL). Cultures treated with equal volumes of solvent (water) were used as controls. At t=24 h, cells cultures were washed with fresh LB by centrifugation at 21,130 g for 3 minutes and diluted 100-fold in 1mL of LB with 1 mM IPTG and 50 µg/mL kanamycin in a test tube. Cells were cultured at 37 ºC for 30 min. At indicated time points (0, 10, 20, and 30 min), cells were pelleted to remove the supernatant and suspended in PBS for analysis by flow cytometry using an LSRII flow cytometer (BD Biosciences, San Jose, CA). Cell populations were identified with forward and side scatter parameters. Using samples collected at t = 0 min, a threshold was set leaving 99 % of the non-fluorescent population to the left (GFP - signal) and anything above this threshold was considered to be signal from new protein. This threshold was applied to samples collected at t = 10, 20 and 30 min. Data plotted correspond to the percentage of the population with GFP + signal at each time point. Samples were excited at 488 nm and fluorescence was collected using a green fluorescene filter (525/50 nm-band-pass filter). Data were acquired and analyzed using the FACSDiVa software and FlowJo, respectively.

**Labeling of PBPs with Bocillin-FL.**  Cultures were grown as described in Supplementary Figure 14. Piperacillin was added at t = 4 h. At 24 h, ten microliters of untreated and piperacillin-treated cultures were taken and transferred to 1.5 mL of cold 90% methanol (before sample). In addition, at 24 h, untreated and piperacillin-treated cultures were washed twice in 2 mL LB, diluted 100-fold in 1 mL LB in a test tube, and incubated at 37 °C with shaking. Following inoculation, at t= 0, 30 and 60 min, 1 mL of culture was collected and transferred to an 1.5 mL Eppendorf tube, cells were harvested by centrifugation at 21,130 g for 3 minutes, 900 µL of the supernatant was removed and pellet resuspended in the remaining 100 µL before transfer to 1.4 mL of cold 90% methanol. These samples were incubated overnight at 4 °C. Next day, cells were harvested by centrifugation at 21,130 g for 3 minutes, washed once in 1 mL of PBS by centrifugation, resuspended in 100 µL of 0.5% Triton X-100, and incubated for 30 min at room temperature. Cells were collected by centrifugation at 21,130 g for 3 minutes, supernatant was removed and cells were washed once in 1 mL of PBS and resuspended in 150 µg/mL of Bocillin-FL in 50 µL of PBS. Labelling was carried out for 10 min at 37 °C followed by centrifugation at 21,130 g for 3 minutes, removal of supernatant, and addition of 1 mL of PBS (this washing procedure was repeated once more). Cells were resuspended in 500 mL PBS for flow cytometry analysis. For determination of the concentration of Bocillin-FL to be used during labeling, the saturating Bocillin-FL concentration was determined by incubating samples, treated as described above, with increasing concentrations of Bocillin-FL (Supplementary Figure 17). For the titration curve, unstained control samples were treated with an equivalent volume of DMSO in 50 µL of PBS. To control for autofluorescence in untreated and piperacillin-treated cells at t= 0, 30 and 60 min, DMSO treated samples (unstained) were used.

**Plasmid DNA supercoiling assay.** Measurements of DNA supercoiling status inside bacterial cells are typically performed using plasmid DNA (10, 11). These experiments are based on the fact that closed circular DNA molecules such as plasmids and chromosomes are negatively supercoiled in *E. coli* (12). Electrophoresis of negatively supercoiled plasmid DNA in an agarose gel containing an intercalating agent that positively supercoils DNA such as chloroquine, generates topoisomers with different levels of supercoiling. These topoisomers are resolved in the gel based on their different migration speeds (11) where fast bands correspond to more supercoiled plasmid DNA and slow bands correspond to less supercoiled or more relaxed DNA. Nicked plasmid DNA runs along with plasmid DNA that does not contain supercoils, and they appear in the gel as a very slow migrating band. We transformed our wild type strain with plasmid DNA, pQE-80L-kan, and carried out treatment with piperacillin or water (untreated control) during transition from exponential to stationary phase (t = 4.5 h, OD_600_ ~ 1). Importantly, we confirmed that transformation of our wild type strain with pQE80L-kan did not impact the capacity of piperacillin to suppress ofloxacin persister formation (Supplementary Figure 18). At t = 24 h, cells were washed to remove piperacillin and diluted into fresh medium. Cultures of MG1655 carrying empty plasmid pQE-80L-kan (2) were grown as described (Supplementary Figure 14). Piperacillin was added at 4.5 h, which is the time when this clone reached OD_600_ ~ 1.0 (similarly grown cultures of MG1655 reach an OD_600_ ~ 1.0 at t = 4 h, the time at which piperacillin treatment was carried out for MG1655). At t = 24 h, untreated and piperacillin-treated cultures were washed in LB twice and diluted (100-fold) in 50 mL of LB containing 50 µg/mL of kanamycin in 500 mL baffled flasks followed by incubation for 5 min at 37 ºC with shaking (250 rpm). Novobiocin was added to LB during washes and 5 min incubation at 60 µg/mL. Because plasmid DNA supercoiling state changes rapidly upon exposure to fresh nutrients (11), treatments were staggered, and samples were processed one at the time alternating the order in which samples were processed in every replicate. Following 5 min incubation, cells were collected by centrifugation (3,220 g for 10 min at room temperature) and plasmids were isolated immediately using Qiaprep Spin miniprep kit (Qiagen). Plasmid DNA was stored at 4 ºC to minimize nicking. Plasmid DNA concentration was determined using the specific DNA binding reagent PicoGreen following the manufacturer’s instructions. Briefly, a standard curve was made with double stranded circular DNA (pQE-80L-kan) of known concentrations. The assay was performed in a black 96-well plate with flat bottom, and fluorescence was measured using a plate reader (Synergy H1 Hybrid Reader, BioTek). Following determination of plasmid DNA concentration, 250 ng of pDNA were run in a 1 % agarose gel in 1X TAE to confirm quality of pDNA preparation and equivalent loadings. The gel was rinsed in water briefly before staining for 1 h in the dark in a solution of 0.5 µg/mL of ethidium bromide dissolved in water. The different topoisomers were resolved in a 1.1 % agarose gel in 1X TAE containing 2.5 µg/mL chloroquine. Agarose gels containing chloroquine are commonly used to measure DNA supercoiling in bacteria (11, 13-15). The solidified gel was transferred to a horizontal electrophoresis chamber and the two reservoirs were filled with 1X TAE buffer containing 2.5 µg/mL of chloroquine (running buffer). The wells in the gel were filled with running buffer and samples of 250 ng of pDNA were loaded using a gel loading dye, Purple 6X (New England Biolabs). The samples were run at 1.5 V/cm for 3 h before the gel was covered with plastic to prevent drying and run for another 18 h at 2 V/cm. Following electrophoresis, the gel was washed extensively for 3 h in 1X TAE followed by rises in water and staining for at least 2 h in an aqueous solution of 0.5 µg/mL of ethidium bromide in the dark. Further overnight destaining of the gel in water was carried out before imaging using Molecular Imager Gel Doc^TM^ XR+ imager (BioRad). Densitometry scans of gel images were generated and analyzed using Image Lab Software (BioRad).

**Protein degradation assay.** Cultures of MO-cured carrying a chromosomally encoded mCherry expression system and pQE-80L plasmid variant with an inducible *ssrA-*tagged *gfp* expression cassette (2) were grown in 2 mL of LB medium with 50 µg/mL kanamycin and 1 mM IPTG and cultured at 37 ºC with shaking (250 rpm). At t= 4h, cells were transferred to 2 mL round-bottom centrifuge-tubes, washed with spent media twice to remove IPTG, and suspended in 2 mL of filter-sterilized spent media (without inducer). Spent media was prepared from wild-type cultures grown under the same conditions. After washing and suspending the cells in spent media, cultures were treated with piperacillin (200 µg/mL). Control cultures were treated with equal volumes of solvent (water). At indicated time points, 100 µL samples were transferred to microcentrifuge tubes with 900 µL PBS and washed twice to remove LB. Cells were diluted to OD_600_ 0.3 in PBS to measure both mCherry and ssrA-tagged-GFP levels in treated and control cultures using a plate reader. Background fluorescence was determined using wild-type cells in PBS at OD_600_ 0.3. To account for the different amount of protein between cells, *ssrA*-tagged GFP levels were normalized to mCherry by dividing *ssrA*-tagged GFP levels by mCherry levels.

**Dissolved oxygen measurements.** The FireStingO_2_ fiber-optic O_2_ meter with the OXYROB 10-CL2 robust oxygen miniprobe (PyroScience, GmbH) was used to measure dissolved oxygen concentrations in cell cultures. Dissolved oxygen levels were reported as the percentage of dissolved oxygen with respect to cell-free media in equilibrium with the atmosphere. After the probe was calibrated with LB media without cells, samples were removed from the shaking incubator and the oxygen probe was inserted into the media at indicated time points. The oxygen levels in all cultures were reported exactly 2 min after insertion of the probe into cultures.

**Microscopy imaging.** Differential interference contrast (DIC) images were taken with a Nikon TE2000 microscope equipped with a 100 x/1.4 numerical aperture objective and Andor Zyla sCMOS camera. Cells from cultures treated with either an inhibitor or an inducer as indicated in Supplementary Figure 14 were collected after treatment (t = 24 h). Untreated cells were used as control. Cells were pelleted by centrifugation (3 min, 21,130 g) from 100 µL culture, resuspended in 100 µl of 4% paraformaldehyde (PFA) and incubated at room temperature for 15 min. After 15 min of incubation, cells were washed with PBS and diluted (10-fold) in the same buffer. Two microliters of cell suspension were loaded onto 1% agarose pads.

**Mass spectrometry.** Cell lysates generated from overnight cultures of MG1655 carrying pKG110-*ftsI*_Trunc_, -*ftsI**_Trunc_, or -*gfp* grown in the presence of 1 mM sodium salicylate and chloramphenicol (for plasmid retention) were loaded into a polyacrylamide gel (16). MG1655 carrying pKG110-*gfp* was used as a negative control. Gel bands were excised at the expected size of FtsI_Trunc_ and FtsI*_Trunc_ (~60 kDa) as described (16) and digested with trypsin (17). Dried gel bands were reconstituted in 0.1% formic acid and analyzed on an Orbitrap Fusion Lumos (ThermoScientific, San Jose, USA) mass spectrometer coupled to an Easy nLC 1200 UPLC and 50 cm long column packed with 1.8 μm C18 particles (Dr. Maisch, Germany). MS/MS data was analyzed in Proteome Discoverer (Thermo Scientific) with 10ppm precursor and 0.4Da fragment ion mass errors using SequestHT and Byonic algorithms against Uniprot *E. coli* database (downloaded 01222018) and appended with common contaminants. Scaffold (version Scaffold_4.4.5, Proteome Software Inc., Portland, OR) was used to validate MS/MS based peptide and protein identifications. Fragments of FtsI_Trunc_ were detected in excised bands from MG1655 carrying pKG110-*ftsI*_Trunc_; fragments of FtsI*_Trunc_, including the Ser307Ala mutation, were detected in excised bands of MG1655 carrying pKG110-*ftsI**_Trunc_; and fragments of FtsI were not detected in excised bands of MG1655 carrying pKG110-*gfp*.
**Statistical analyses.** Two-tailed t-tests with unequal variances were used for statistical comparisons and p-values ≤ 0.05 were considered significant. For cell division, protein synthesis, and RNA integrity results, asterisks indicated statistically-significant differences between the sample and control groups. For relative ATP, relative protein, and relative RNA abundances, asterisks designated statistically-significant differences from a mean value of 1. For survival and growth resumption data, asterisks indicated statistically-significant differences between the log-transformed values of results from sample and control groups, because those results were obtained from sequential dilution series.

**References**

1. Orman MA, Brynildsen MP. Dormancy is not necessary or sufficient for bacterial persistence. Antimicrob Agents Chemother. 2013;57(7):3230-9.

2. Orman MA, Brynildsen MP. Inhibition of stationary phase respiration impairs persister formation in *E. coli*. Nat Commun. 2015;6:7983.

3. Baba T, Ara T, Hasegawa M, Takai Y, Okumura Y, Baba M, et al. Construction of *Escherichia coli* K-12 in-frame, single-gene knockout mutants: the Keio collection. Molecular systems biology. 2006;2:2006 0008.

4. Shiomi D, Margolin W. Dimerization or oligomerization of the actin-like FtsA protein enhances the integrity of the cytokinetic Z ring. Mol Microbiol. 2007;66(6):1396-415.

5. Yen KM. Construction of cloning cartridges for development of expression vectors in gram-negative bacteria. J Bacteriol. 1991;173(17):5328-35.

6. Wissel MC, Wendt JL, Mitchell CJ, Weiss DS. The transmembrane helix of the *Escherichia coli* division protein FtsI localizes to the septal ring. J Bacteriol. 2005;187(1):320-8.

7. Andrews JM. Determination of minimum inhibitory concentrations. Journal of Antimicrobial Chemotherapy. 2001;48:5-16.

8. Ferullo DJ, Cooper DL, Moore HR, Lovett ST. Cell cycle synchronization of *Escherichia coli* using the stringent response, with fluorescence labeling assays for DNA content and replication. Methods. 2009;48(1):8-13.

9. Akerlund T, Nordstrom K, Bernander R. Analysis of cell size and DNA content in exponentially growing and stationary-phase batch cultures of *Escherichia coli*. J Bacteriol. 1995;177(23):6791-7.

10. Levene SD. Analysis of DNA topoisomers, knots, and catenanes by agarose gel electrophoresis. Methods Mol Biol. 2009;582:11-25.

11. Liu Y, Hua ZC, Leng F. DNA Supercoiling Measurement in Bacteria. Methods Mol Biol. 2018;1703:63-73.

12. Irobalieva RN, Fogg JM, Catanese DJ, Jr., Sutthibutpong T, Chen M, Barker AK, et al. Structural diversity of supercoiled DNA. Nat Commun. 2015;6:8440.

13. Yang J, Annamalai T, Cheng B, Banda S, Tyagi R, Tse-Dinh YC. Antimicrobial Susceptibility and SOS-Dependent Increase in Mutation Frequency Are Impacted by *Escherichia coli* Topoisomerase I C-Terminal Point Mutation. Antimicrob Agents Chemother. 2015;59(10):6195-202.

14. Clark DJ, Leblanc BP. Analysis of DNA Supercoiling Induced by DNA-Protein Interactions. Methods Mol Biol. 2015;1334:161-72.

15. Valjavec-Gratian M, Henderson TA, Hill TM. Tus-mediated arrest of DNA replication in *Escherichia coli* is modulated by DNA supercoiling. Mol Microbiol. 2005;58(3):758-73.

16. Henry TC, Brynildsen MP. Development of Persister-FACSeq: a method to massively parallelize quantification of persister physiology and its heterogeneity. Sci Rep. 2016;6:25100.

17. Shevchenko A, Tomas H, Havlis J, Olsen JV, Mann M. In-gel digestion for mass spectrometric characterization of proteins and proteomes. Nat Protoc. 2006;1(6):2856-60.
