# Supplementary material for: Stationary phase persister formation in Escherichia coli can be suppressed by piperacillin and PBP3 inhibition
Source: BMC Microbiol. 2019 Jun 24;19:140. doi: 10.1186/s12866-019-1506-7 (PMC6591824; doi:10.1186/s12866-019-1506-7)
Supplement: Supplementary file 16 — Table S1. Bacterial strains and plasmids. (DOCX 24 kb) [file 12866_2019_1506_MOESM16_ESM.docx]

| **Strain** | **Relevant genotype** | **Source** |
| --- | --- | --- |
| MG1655 | F-, λ-, *ilvG*-, *rfb-50, rph-1* | \| ATCC 700926 ^1^ \| \| --- \| |
| MO001 | Δ*lacZYA::*P_lacIQ_-P_T5_*-mCherry,* kan^R^ | ^2^ |
| MO-cured | Δ*lacZYA::*P_lacIQ_-P_T5_*-mCherry* | ^3^ |
| MG1655Δ*dpiA*::kan | Δ*dpiA*::kan | Generated by P1 transduction of Keio mutant ^4^ into MG1655 |
| **Plasmid** | **Description** | **Source** |
| pWM2765 | *ftsZ* in pKG110. pKG110 is a pACYC184 derivative containing the *nahG* promoter and *nahR* regulator.  Contains a chloramphenicol resistance marker. | ^5^ |
| pKG110-*ftsI* | pWM2765 in which *ftsZ* was replaced by *ftsI* | This study |
| pKG110-*ftsI** | pWM2765 in which *ftsZ* was replaced by *ftsI** (Ser3017Ala) | This study |
| pKG110-*ftsI*_Trunc_ | pKG110-*ftsI* in which *ftsI* was replaced by *ftsI*_Trunc_ | This study |
| pKG110-*ftsI**_Trunc_ | pKG110-*ftsI** in which *ftsI** was replaced by *ftsI**_Trunc_ | This study |
| pQE-80L-kan | pQE-80L variant containing conferring kanamycin resistance | ^3^ |
| pQE-80L-*gfp* | *gfp* in pQE-80L variant conferring kanamycin resistance | ^3^ |
| pQE-80L-*gfp-ssrA* | *gfp-ssrA* in pQE-80L conferring kanamycin resistance | ^3^ |

**Supplementary Table 1. Bacterial strains and plasmids**

**References**

1. Kohanski MA, Dwyer DJ, Hayete B et al. A common mechanism of cellular death induced by bactericidal antibiotics. *Cell* 2007; **130**: 797-810.

2. Orman MA, Brynildsen MP. Dormancy is not necessary or sufficient for bacterial persistence. *Antimicrob Agents Chemother* 2013; **57**: 3230-9.

3. Orman MA, Brynildsen MP. Inhibition of stationary phase respiration impairs persister formation in *E. coli*. *Nat Commun* 2015; **6**: 7983.

4. Baba T, Ara T, Hasegawa M et al. Construction of *Escherichia coli* K-12 in-frame, single-gene knockout mutants: the Keio collection. *Mol Syst Biol* 2006; **2**: 2006 0008.

5. Shiomi D, Margolin W. Dimerization or oligomerization of the actin-like FtsA protein enhances the integrity of the cytokinetic Z ring. *Mol Microbiol* 2007; **66**: 1396-415.
