# Supplementary material for: Stationary phase persister formation in Escherichia coli can be suppressed by piperacillin and PBP3 inhibition
Source: BMC Microbiol. 2019 Jun 24;19:140. doi: 10.1186/s12866-019-1506-7 (PMC6591824; doi:10.1186/s12866-019-1506-7)
Supplement: Supplementary file 17 — Table S2. DNA oligonucleotides. (DOCX 17 kb) [file 12866_2019_1506_MOESM17_ESM.docx]

**Supplementary Table 2. DNA oligonucleotides**

| **Primers used to generate *ftsI* and *ftsI** expression plasmids** | | |
| --- | --- | --- |
| **Primer name** | **Sequence** | **Description** |
| nahR_MfeI_Fwd | GCCAATTGGGGGCCTGGTGAGC | Used in conjunction with Pg(nahR)_ftsI_Rev to amplify promoter sequence for cross-over PCR with either *ftsI* or *ftsI** PCR product.  Used in conjunction with ftsI-Rev (XhoI) to amplify either *ftsI* or *ftsI** cloning insert containing promoter sequence |
| ftsI-Rev (XhoI) | CCCTCGAGTTACGATCTGCCACCTGTCC | Used in conjunction with nahR_MfeI_Fwd to amplify either *ftsI* or *ftsI** cloning insert containing promoter sequence  Used in conjunction with ftsI_Ser307Ala_Fwd to amplify one PCR fragment for cross-over PCR to generate *ftsI** |
| ftsI_Ser307Ala_Fwd | GACGTGTTTGAACCGGGCGCAACGGTTAAACCGATGGTGGTA | Used in conjunction with ftsI-Rev (XhoI) to amplify one PCR fragment for cross-over PCR to generate *ftsI** |
| ftsI_Ser307Ala_Rev | TACCACCATCGGTTTAACCGTTGCGCCCGGTTCAAACACGTC | Used in conjunction with Pg(nahR)_ftsI_Fwd to amplify one PCR fragment for cross-over PCR to generate *ftsI** |
| Pg(nahR)_ftsI_Rev | GTTTTCGCCGCTGCTTTCATGTACTCGTGATGGCTTTATT | Used in conjunction with nahR_MfeI_Fwd to amplify promoter sequence for cross-over PCR with either *ftsI* or *ftsI** PCR product |
| Pg(nahR)_ftsI_Fwd | AATAAAGCCATCACGAGTAATGAAAGCAG CGGCGAAAAC | Used in conjunction with ftsI_Ser307Ala_Rev to amplify one PCR fragment for cross-over PCR to generate either *ftsI**.  Used in conjunction with ftsI-Rev (XhoI) to amplify either *ftsI* or *ftsI** fragment for cross-over PCR with promoter sequence |
| **Primers used in chromosomal *dpiA* perturbation** | | |
| **Primer name** | **Sequence** | **Description** |
| dpiA_Ext_Fwd | ATTGCCAGCTACGTAACGC | Used in conjunction with KanR_Rev to confirm chromosomal location of kanamycin cassette |
| dpiA_Int_Fwd | ACATTCCCGGATTCAGTCAG | Used in conjunction with dpiA_Int_Rev to rule out gene duplication after deletion of *dpiA* gene |
| dpiA_Int_Rev | GCTTCAGACACCGTTTCCAT | Used in conjunction with dpiA_Int_Fwd to rule out gene duplication after deletion of *dpiA* gene |
| KanR_Rev | ATGATGGATACTTTCTCGGCAGGAG | Used in conjunction with dpiA_Ext_Fwd to confirm chromosomal location of kanamycin cassette |
| **Primers used to generate *ftsI*_Trunc_ and *ftsI**_Trunc_ expression plasmids** | | |
| **Primer name** | **Sequence** | **Description** |
| pKG110-*ftsI*_Trunc__Fwd | AATAAAGCCATCACGAGTACATGATCTCCCCGGATATGCTGGT | Used in conjunction with pKG110-Rev to assemble plasmid pKG110 harboring truncated FtsI/* using Gibson assembly |
| pKG110-*ftsI*_Trunc__Rev | TTTATCCGCGGATCCTCGAGTTACGATCTGCCACCTGTC | Used in conjunction with pKG110-Fwd to assemble plasmid pKG110 harboring truncated FtsI/* using Gibson assembly |
| pKG110-Fwd | CTCGAGGATCCGCGGATAAA | Used in conjunction with pKG110-*ftsI*_Trunc__Rev to assemble plasmid harboring truncated FtsI/* using Gibson assembly |
| pKG110-Rev | GTACTCGTGATGGCTTTATTG | Used in conjunction with pKG110-*ftsI*_Trunc__Fwd to assemble plasmid harboring truncated FtsI/* using Gibson assembly |
| **Primers used to sequence *ftsI*, *ftsI**, *ftsI*_Trunc_, and *ftsI**_Trunc_ in pGK110** | | |
| **Primer name** | **Sequence** | **Description** |
| pKG110-Seq_Fwd | GACCAGCAACTGGTTGAACA | Used to confirm, by Sanger sequencing, the correct sequence of cloned genes in pKG110 |
| pKG110-Seq_Rev | TGCTTGCTGTTCTTGAATGG | Used to confirm, by Sanger sequencing, the correct sequence of cloned genes in pKG110 |
| FtsI_Int_Fwd | TGAGGGCGTTGAGAAGAGTT | Used to confirm, by Sanger sequencing, the correct sequence of cloned *ftsI* or *ftsI** genes |
| FtsI_Int_Rev | TCGCGCTAACTGTAATGGTG | Used to confirm, by Sanger sequencing, the correct sequence of cloned *ftsI* or *ftsI** genes |
